# Analysis of a Plant Complex Resistance Gene Locus Underlying Immune-Related Hybrid Incompatibility and Its Occurrence in Nature

**DOI:** 10.1371/journal.pgen.1004848

**Published:** 2014-12-11

**Authors:** Rubén Alcázar, Marcel von Reth, Jaqueline Bautor, Eunyoung Chae, Detlef Weigel, Maarten Koornneef, Jane E. Parker

**Affiliations:** 1 Department of Natural Products, Plant Biology and Soil Science, Laboratory of Plant Physiology, Faculty of Pharmacy. University of Barcelona, Barcelona, Spain; 2 Department of Plant-Microbe Interactions, Max Planck Institute for Plant Breeding Research, Cologne, Germany; 3 Department of Plant Breeding and Genetics, Max Planck Institute for Plant Breeding Research, Cologne, Germany; 4 Department of Molecular Biology, Max Planck Institute for Developmental Biology, Tübingen, Germany; 5 Cluster of Excellence on Plant Sciences (CEPLAS), Germany; Virginia Tech, United States of America

## Abstract

Mechanisms underlying speciation in plants include detrimental (incompatible) genetic interactions between parental alleles that incur a fitness cost in hybrids. We reported on recessive hybrid incompatibility between an *Arabidopsis thaliana* strain from Poland, Landsberg *erecta* (L*er*), and many Central Asian *A. thaliana* strains. The incompatible interaction is determined by a polymorphic cluster of Toll/interleukin-1 receptor-nucleotide binding-leucine rich repeat (TNL) *RPP1* (*Recognition of Peronospora parasitica1*)-like genes in L*er* and alleles of the receptor-like kinase *Strubbelig Receptor Family 3* (*SRF3*) in Central Asian strains Kas-2 or Kond, causing temperature-dependent autoimmunity and loss of growth and reproductive fitness. Here, we genetically dissected the *RPP1*-like L*er* locus to determine contributions of individual *RPP1*-like L*er* (*R1*–*R8*) genes to the incompatibility. In a neutral background, expression of most *RPP1*-like L*er* genes, except *R3*, has no effect on growth or pathogen resistance. Incompatibility involves increased *R3* expression and engineered *R3* overexpression in a neutral background induces dwarfism and sterility. However, no individual *RPP1*-like L*er* gene is sufficient for incompatibility between L*er* and Kas-2 or Kond, suggesting that co-action of at least two *RPP1*-like members underlies this epistatic interaction. We find that the *RPP1*-like L*er* haplotype is frequent and occurs with other L*er RPP1*-like alleles in a local population in Gorzów Wielkopolski (Poland). Only Gorzów individuals carrying the *RPP1*-like L*er* haplotype are incompatible with Kas-2 and Kond, whereas other *RPP1*-like alleles in the population are compatible. Therefore, the *RPP1*-like L*er* haplotype has been maintained in genetically different individuals at a single site, allowing exploration of forces shaping the evolution of *RPP1*-like genes at local and regional population scales.

## Author Summary

In plants, naturally evolving disease resistance (*R*) genes can cause autoimmunity when combined with different genetic backgrounds. This phenomenon, called immune-related hybrid incompatibility (HI), leads to growth inhibition and fitness loss due to inappropriate activation of defense. HI likely reflects different evolutionary paths of immune-related genes in nature. We have examined the genetic architecture of a complex *R* locus present in a Central European accession (L*er*) which underlies HI with Central Asian accessions of *Arabidopsis*. We show that expression of one gene (*R3*) within the L*er* cluster of eight tandem *R* genes (*R1–R8*) controls the balance between growth and defense but that *R3* needs at least one other co-acting member within the *R* locus to condition HI. We traced the *R1–R8* haplotype to a local population of L*er* relatives in Poland where it also underlies HI with Central Asian accessions. Occurrence of the incompatible haplotype in ∼30% of genetically diverse local individuals, suggests that it has not arisen recently and has been maintained through selection or drift. Co-occurrence in the same population of individuals containing different *R* genes that do not cause HI provides a basis for determining genetic and environmental forces influencing how plant immunity genes evolve and diversify.

## Introduction

Understanding the processes by which new species arise is an important evolutionary question [1]. In plants, polyploidy is one of the best known mechanisms in speciation, together with other pre-zygotic and post-zygotic barriers [2]. However, more discrete and often cumulative changes in plant genomes can lead to reproductive barriers and, potentially, isolation [3]. Intrinsic to speciation is the divergence of populations, which allows accumulation of genetic differences as a result of drift, local adaptation or coevolution. Such evolutionary processes may create novel alleles or genes that, when combined with other forms from divergent populations, cause hybrid failure to various degrees [3], [4]. These alleles and the resulting hybrids are referred to as ‘incompatible’ and they have been documented in plant breeding programs e.g. [5], although incompatibilities are not limited to crops, as demonstrated by their occurrence in *Arabidopsis thaliana* [6] and *Mimulus guttatus* [7] populations.

In some genetically recessive hybrid incompatible (HI) interactions, parental lineages may have experienced alternate loss-of-function of duplicated genes required for viability as a result of relaxed purifying selection [8], [9]. Other more complex dominant and recessive hybrid incompatibilities in plants involve allelic mismatches between immune-related genes that trigger constitutive activation of defenses in the absence of pathogen challenge. Hybrid necrosis is often a symptom of resistance deregulation and its cost on growth and reproduction [10].

Plants are frequently attacked by microbial pathogens which cause disease by deploying virulence factors (effectors) that interfere with plant host defenses [11]. Pathogen effectors are in turn recognized, directly or indirectly, by intracellular nucleotide binding-leucine rich repeat (NLR) receptors to induce effector-triggered immunity, which is a rapid host cellular resistance response often associated with localized programmed cell death [11]. NLRs broadly fall into two structural sub-classes carrying either an N-terminal Toll/Interleukin1-receptor domain (known as TNLs) or a coiled-coil domain (CNLs). Consistent with their role as sensors at the molecular interface with rapidly evolving pathogen effector arsenals, there has been massive expansion and diversification of *NLR* gene families across plant lineages [12], [13]. Nevertheless, the rate of *NLR* variation is unlikely to keep pace with microbial change, and therefore maintenance of diverse *NLR* alleles within a population might be an important determinant of host-pathogen coevolution [14]. Accordingly, some *TNL* genes exhibit molecular signatures consistent with patterns of balancing selection [14]–[16] which would contribute to the standing genetic variation present in nature. Further evidence suggests that plants also extend their resistance spectrum by intercepting actions of multiple effectors on a limited set of cellular targets [17]. There are examples of NLRs monitoring or ‘guarding’ the status of an effector target (the guardee) [11]. Maintaining appropriate ‘guard-guardee’ associations while tolerating NLR variation presents a challenge because even small molecular rearrangements might disturb NLR homeostasis, leading to autoimmunity and impaired growth. Such mismatches would be particularly exposed in interactions in which protein pairs have diverged independently in populations and natural variants arisen that mimic effector modifications [18].

*TNLs* are highly polymorphic genes in *Arabidopsis* and many reside in clusters [12], [19]. Their characteristic three-domain composition and within-gene sequence repetition can further promote high levels of polymorphism through non-allelic homologous recombination [12], [20], [21] and *TNLs* are among plant genes with the highest naturally occurring sequence variation, closely followed by receptor-like kinase (*RLK*) genes [9], [12], [22].

Several cases have been reported in which autoimmunity results from allelic interactions involving *TNL* or *RLK* genes. Intraspecific incompatibilities in *Arabidopsis* involve interactions between *TNL* genes [6], *TNLs* with *RLKs* [23], or *TNLs* with a gene encoding a cysteine biosynthetic enzyme [24]. Interspecific HI between crossable species has been reported to involve the CNL receptor-guarded effector target *RIN4* in lettuce [25] and *NLR*/*RLK* combinations in rice [26], [27]. The nature of the epistatic interaction is therefore not entirely predictable, although the frequency with which a polymorphic cluster of *NLR* and/or *RLK* genes is involved likely reflects the occurrence of high genetic variation at these loci, or lower phenotypic buffering capacity for *NLR* and/or *RLK* variation [12], [28]. In *Arabidopsis* accession Ws-0, *TNL* genes within the *Recognition of Peronospora parasitica1* (*RPP1*) resistance locus confer specific resistance to infectious downy mildew *Hyaloperonospora arabidopsidis* (*Hpa*) isolates (formerly *Peronospora parasitica*) [29], [30]. Interestingly, an *Arabidopsis RPP1*-like locus with no known pathogen recognition specificity underlies three independent autoimmune interactions [6], [24], [31], two of which involve the *RPP1*-like Landsberg *erecta* (L*er*) haplotype [24], [31]. This suggests that the *RPP1*-like locus is predisposed to immune-related hybrid incompatibility in this species.

We reported the occurrence of HI between L*er* and individuals from Central Asian populations (Kas-2 and Kond). Incompatible L*er*/Kas-2 and L*er*/Kond hybrids exhibit dwarfism and sterility at moderately low temperature (14–16°C) typical for ambient temperatures during the natural growing season of many *A. thaliana* accessions, and these phenotypes are suppressed at higher temperature (20–22°C) [23], [31]. L*er*/Kas-2 and L*er*/Kond incompatibilities are caused by a common recessive genetic interaction between the *RPP1*-like L*er* locus and Kas-2 or Kond alleles of *RLK Strubbelig Receptor Family 3* (*SRF3*). The *RPP1*-like locus in L*er* contains eight tandemly arranged *TNL* genes (*R1*–*R8*, including *R6* which is a truncated form). Col-0 has only two *RPP1*-like genes at this locus (*At3g44630* and *At3g44670*), similar to its close relative *Arabidopsis lyrata* [32], suggesting that L*er* carries a derived *RPP1*-like haplotype. An *RPP1*-like structural variant in accession Uk-1 likely triggers incompatibility with Uk-3, an accession from the same local population [6]. Strikingly, different genetic determinants underlie the *RPP1*-like Uk-1 and L*er* incompatibilities [31]. The Uk-1 *RPP1*-like locus is incompatible with a Uk-3 allelic form of the *TNL* gene, *SSI4* (*suppressor of salicylic acid insensitivity of npr1-4*) [6], but not with Kas-2 or Kond alleles of *SRF3* [31]. Hence, independent epistatic networks underlie these incompatibilities.

Incompatible *SRF3* alleles are frequently found in Central Asia and exhibit molecular patterns consistent with signatures of a recent selective sweep, suggesting that incompatibility with L*er* has arisen as a by-product of selection [23]. By contrast, little is known about the natural distribution of the *RPP1*-like L*er* haplotype in Central Europe or the potential benefit or cost of carrying it. Here, we have examined the genetic basis of the *RPP1*-like L*er* incompatibility and the occurrence of the *RPP1*-like L*er* haplotype worldwide. We find that it has been maintained in a local Central European natural population over at least seven decades. We tested for involvement of individual *RPP1*-like L*er* genes in HI between L*er* and Kas-2 using artificial microRNA (amiRNA) silencing and analysis of *RPP1*-like L*er* transgenes in neutral or incompatible *Arabidopsis* backgrounds. We establish that individual *RPP1*-like L*er* genes contribute differently to the trade-off between growth and disease resistance. Incompatibility between L*er* and Kas-2 is associated with higher expression of the *RPP1*-like *R3* gene and engineered *R3* over expression causes autoimmunity. However, individual *RPP1*-like L*er* gene members are insufficient to condition HI with Kas-2 and Kond. We conclude that a minimum expression threshold of two or more *RPP1*-like L*er* genes in combination with Kas-2 or Kond *SRF3* allelic forms is required for autoimmunity and HI. Finally, we show that the incompatible *RPP1*-like L*er* haplotype is frequent in a local Central European population where it co-occurs with other *RPP1*-like genes not triggering incompatibility with Kas-2 or Kond. Our study reveals the complex nature and local genetic diversity of the *RPP1*-like L*er* locus underlying incompatibility with Central Asian populations of *Arabidopsis*.

## Results

### amiRNA silencing of *RPP1*-like L*er* genes

**Figure 1:**
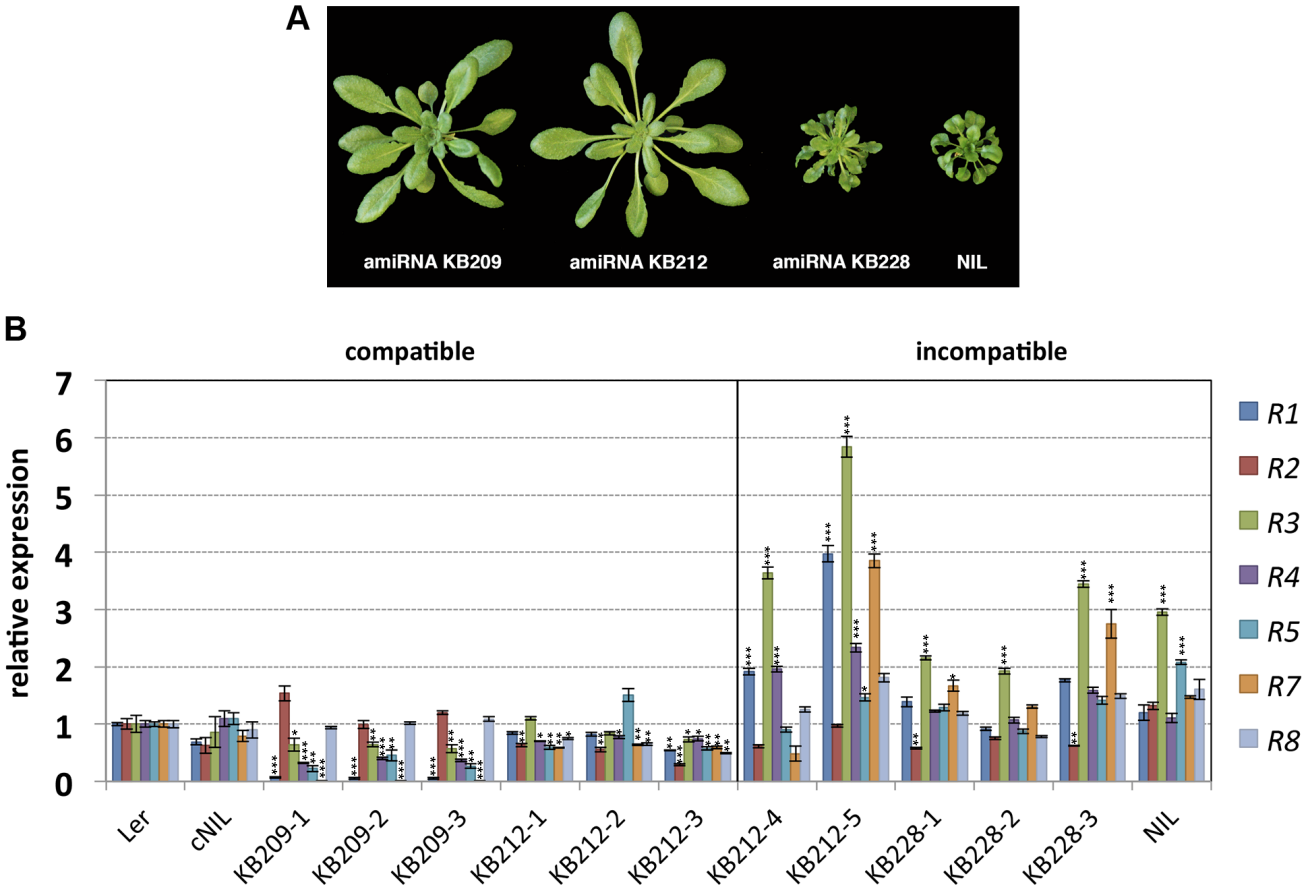
Growth phenotype of amiRNA lines at 14–16°C and *RPP1*-like gene expression. (**A**) Growth phenotype of 5-week old L*er*/Kas-2 NIL plants transformed with amiRNAs effective (KB209 and KB212) or not effective (KB228) suppressing incompatibility. (**B**) Expression of individual *RPP1*-like L*er* genes determined by qRT-PCR in suppressed (compatible) amiRNA KB209 lines (KB209-1, KB209-2, KB209-3), KB212 (KB212-1, KB212-2, KB212-3) and non-suppressed (incompatible) amiRNA lines KB212 (KB212-4, KB212-5), KB228 (KB228-1, KB228-2, KB228-3). Values are relative to L*er* and the mean ± SD of three biological replicates each using three technical replicates. cNIL (complemented NIL), NIL (incompatible L*er*/Kas-2 near-isogenic line) [23], [31]. Significant differences in gene expression between L*er* and other genotypes using Student's t-test are indicated by asterisks: *P<0.05, **P<0.01, ***P<0.005.

Previously, we mapped the L*er* locus causing incompatibility with Kas-2 and Kond Central Asian accessions (*SRF3* forms) to a large ∼87 kb cluster of *RPP1*-like genes on chromosome 3 [31]. We also established that HI was suppressed by loss-of-function mutations of the TNL immunity regulator *EDS1* or by depletion of the defense signaling hormone salicylic acid (SA), consistent with *TNL* genes driving HI [31]. To ascertain whether HI is due to one or more *RPP1*-like L*er* genes within the locus, we used artificial microRNA (amiRNA) silencing of an incompatible L*er*/Kas-2 near isogenic line (NIL) which contains a L*er* introgression spanning the *RPP1*-like locus in a Kas-2 background [31]. Incompatible NIL plants were transformed with amiRNAs KB209, KB212 and KB228 originally designed against *RPP1*-like genes in Uk-1 (**S1 Table**). Of the three amiRNAs used, only KB209 and KB212 have predicted complementarity with *RPP1*-like L*er* genes. Multiple independent NIL lines transformed with amiRNAs KB209, KB212 and KB228 (the latter used as a negative control) were tested for suppression of incompatible phenotypes at 14–16°C. We observed suppression of incompatibility in all NIL plants transformed with amiRNA KB209, and in most KB212 transformants (**Fig. 1A**). As expected, KB228 did not rescue the incompatible NIL phenotype (**Fig. 1A**).

Using quantitative RT-PCR (qRT-PCR) with primer pairs that discriminated between individual *RPP1*-like L*er* genes (S2 Table), we determined expression of each gene within the *RPP1*-like L*er* cluster in the amiRNA lines with and without HI suppression, in the original NIL background, a complemented NIL line (cNIL; transformed with the compatible *SRF3* L*er* allele [23]), and the L*er* parental accession (**Fig. 1B**). Quantitative expression analyses indicated silencing of most *RPP1*-like L*er* genes by amiRNAs KB209 and KB212 in suppressed lines (compatible), except for *R2* and *R8*, which were expressed at wild-type (L*er*) levels in KB209 lines (**Fig. 1B**). These results narrowed the potential *RPP1*-like incompatibility determinants down to *R1*, *R3*, *R4*, *R5* and *R7*. In non-suppressed incompatible lines (KB228, KB212-4 and KB212-5) and the NIL, we consistently detected higher *RPP1*-like *R3* expression levels (≥2-fold) compared to L*er*, the cNIL and compatible amiRNA lines (**Fig. 1B**). These results show that loss of HI correlates with reduced expression of multiple *RPP1*-like L*er* genes, whereas maintenance of HI is associated with enhanced expression of *R3*. Thus, *R3* might be a key factor in the incompatibility with Kas-2.

### Contribution of *RPP1*-like L*er* genes to the trade-off between growth and disease resistance

**Figure 2:**
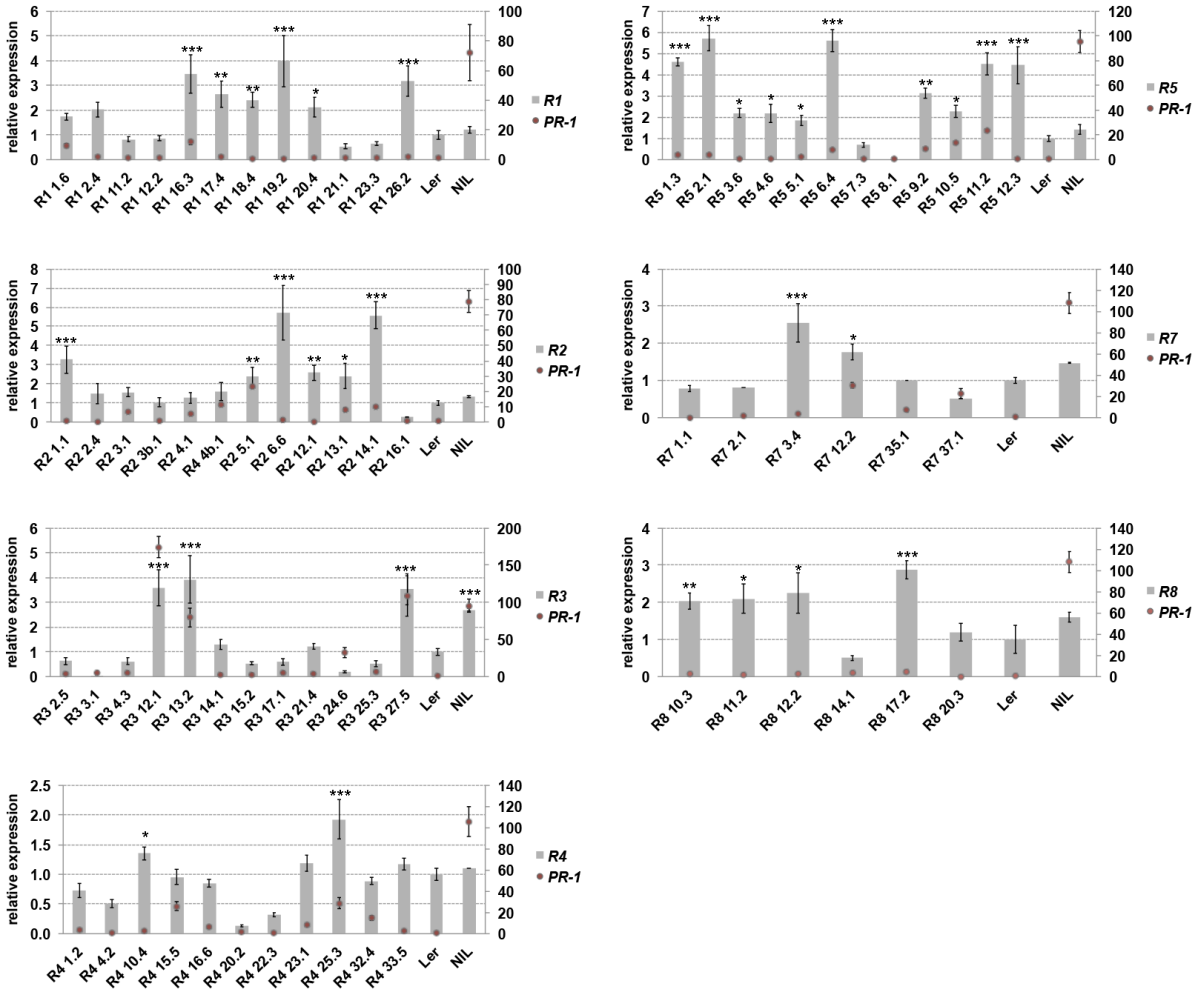
Transgene and *PR-1* expression in Col*^RPP1^* lines. Expression of *RPP1*-like L*er R1*, *R2*, *R3*, *R4*, *R5*, *R7* and *R8* transgenes (left axis) and *PR-1* (right axis) determined in individual homozygous Col*^RPP1^* lines, L*er* and NIL plants grown at 14–16°C by qRT-PCR. Values are relative to L*er* and the mean ± SD of three biological replicates each using three technical replicates. NIL (incompatible L*er*/Kas-2 near-isogenic line [31]). Significant differences in gene expression between L*er* and different Col*^RPP1^* lines using Student's t-test are indicated by asterisks: *P<0.05, **P<0.01, ***P<0.005.

Prolonged activation of defenses bears a fitness cost for the plant [33], [34] and this might shape the genetic composition of *Resistance* (*R*) genes in natural populations [13]. We measured the contribution of individual *RPP1*-like L*er* genes to the trade-off between growth and disease resistance by transforming a neutral (compatible) background, accession Col-0, with genomic constructs of each *RPP1*-like L*er* gene under control of its native 5′ and 3′ sequences. These lines are referred to as Col*^RPP1Ler^*. We also included *RPP1*-like L*er R1* and *R5* genes, which contain stop codons in their coding sequences (**S1 Figure**). Expression of the *RPP1*-like L*er* transgenes in Col-0 was detectable and ranged from 0.5 to 5.5-fold their native expression levels in L*er* (**Fig. 2**). Interestingly, Col*^RPP1Ler^ R3* lines with higher expression (lines 12, 13 and 27) exhibited dwarfism and sterility at 14–16°C, which were suppressed at 20–22°C (**S2 Figure**). By contrast, Col*^RPP1Ler^ R1*, *R2*, *R4*, *R5*, *R7* and *R8* lines did not show obvious growth defects at 14–16°C regardless of the transgene expression level (**Fig. 2** **and S3 Figure**). Expression of the defense marker gene *PR-1* was used to monitor defense activation in the different transgenic lines (**Fig. 2**). Col*^RPP1^* ^L*er*^ *R3* lines with higher transgene expression also exhibited high *PR-1* expression. Expression of *PR-1* remained low in Col*^RPP1^* ^-like L*er*^ *R1*, *R2*, *R4*, *R5*, *R7* and *R8* lines and variation in *PR1* transcript levels between lines did not correlate with transgene expression (**Fig. 2**). Cell death lesions were detected in leaves of the stunted Col*^RPP1^* ^L*er*^ *R3* lines at 14–16°C, as observed previously for L*er*/Kas-2 incompatible lines [31], but were absent in all other Col*^RPP1^* ^-like L*er*^ *R1*, *R2*, *R4*, *R5*, *R7* and *R8* lines grown under the same conditions (**S4 Figure**).

We tested the different Col*^RPP1^* lines for basal immunity to the virulent *Hpa* isolate Noco2 (**S5 Figure**) [23], and for *TNL* (*RPP2*)-mediated immunity to avirulent *Hpa* isolate Cala2 (**S6 Figure**). Effector-triggered *TNL* immunity is often accompanied by a hypersensitive response (HR) involving localized plant cell death at infection sites [35]. These pathology assays showed that *RPP1*-L*er R1*, *R2*, *R4*, *R5*, *R7* or *R8* genes did not alter Col basal disease resistance against *Hpa* Noco2 (**S5 Figure**) or *TNL* resistance against *Hpa* Cala2 (**S6 Figure**). The results argue against a contribution of individual *RPP1*-like L*er R1*, *R2*, *R4*, *R5*, *R7* or *R8* to defense, as measured by their *Hpa* disease resistance phenotypes. However, overexpression of *RPP1*-like L*er R3* in Col-0 caused increased cell death in response to virulent *Hpa* isolate Noco2 (**S5 Figure**) and extended HR-like lesioning in response to avirulent *Hpa* Cala2 (**S6 Figure**). These infection phenotypes are similar to those observed in incompatible L*er*/Kas-2 lines [31]. Basal and *TNL* triggered resistance phenotypes were not altered in transgenic lines with lower *R3* expression (**S7 Figure**). In summary, the results show that overexpression of *RPP1*-like L*er R3* causes HI-like autoimmunity and suggest that *RPP1*-like L*er R3* expression affects the trade-off between plant growth and disease resistance.

### Effects of *RPP1*-like L*er R3* expression with different allelic combinations at *SRF3* and QTL5

**Figure 3:**
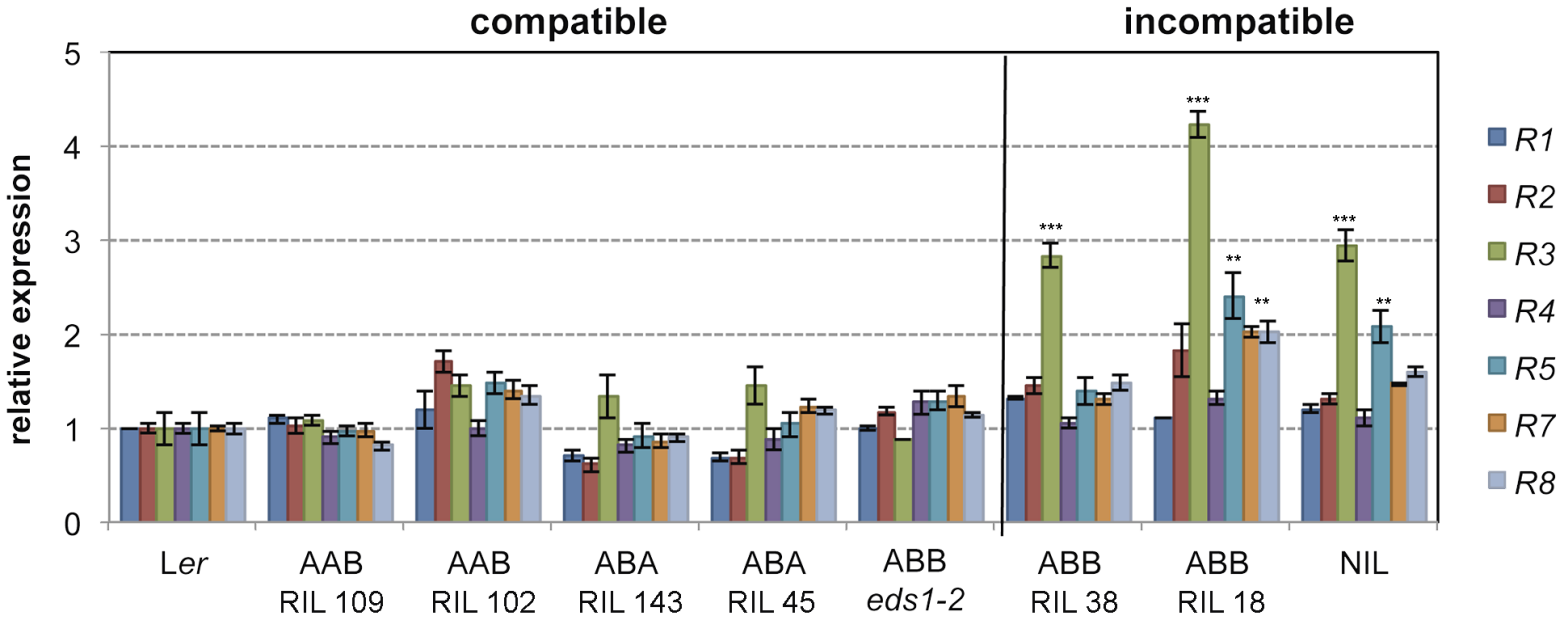
Effects of *R3* expression with different allelic combinations at *SRF3* and QTL5. Expression of *RPP1*-like L*er* genes determined by qRT-PCR in different L*er*/Kas-2 recombinant inbred lines (RIL) carrying homozygous allelic combinations at *RPP1*-like/*SRF3*/QTL5 loci: L*er*/L*er*/Kas-2 (*AAB*), L*er*/Kas-2/L*er* (*ABA*), L*er*/Kas-2/Kas-2 (*ABB*). *AAB* and *ABA* represent compatible allele combinations, whereas *ABB* triggers incompatibility. The *ABB eds1-2* line carrying an *EDS1* loss-of-function mutation is described in [31]. Values are relative to L*er* and the mean ± SD of three biological replicates each using three technical replicates. Significant differences in the gene expression between L*er* and different genotypes using Student's t-test are indicated by asterisks: *P<0.05, **P<0.01, ***P<0.005.

The *RPP1*-like L*er* locus is incompatible with homozygous Kas-2 alleles at *SRF3* and a third locus (QTL5) involved in HI with Kas-2 [31]. Because *RPP1*-like L*er R3* overexpression causes enhanced immunity in a neutral background, we asked whether allelic variation at the *SRF3* and QTL5 interacting loci affect expression of genes within the *RPP1*-like L*er* locus. For this, we measured expression of individual *RPP1*-like L*er* genes in L*er*/Kas-2 recombinant inbred lines (RILs) [36] carrying incompatible (*RPP1*-like/*SRF3*/QTL5: L*er*/Kas-2/Kas-2) or compatible combinations of alleles at *RPP1*, *SRF3* and QTL5 (L*er*/L*er*/Kas-2 and L*er*/Kas-2/*Ler*) (**Fig. 3**). Expression analyses showed that *RPP1*-like L*er R3* transcript levels were significantly (≥2.0-fold) higher in RILs carrying incompatible alleles (**Fig. 3**). Also, *R3* transcriptional up-regulation did not occur in an incompatible L*er*/Kas-2 line carrying the *eds1-2* mutation (**Fig. 3**). These results underscore the relationship between incompatibility and *RPP1*-like L*er R3* expression inferred from the amiRNA analysis (**Fig. 1B**), and suggest that incompatibility involves *EDS1*-dependent upregulation of *RPP1*-like L*er R3*.

### Contribution of individual *RPP1*-like L*er* genes to incompatibility

We then examined the contribution of individual *RPP1*-like L*er* genes to incompatibility with Kas-2 and Kond, by crossing these accessions with multiple independent Col*^RPP1^* transgenic lines differing in *RPP1*-like L*er* transgene expression (**S3 Table**). We generated and scored an average of 220 F_2_ plants per population for the segregation of HI phenotypes at 14–16°C. As expected, progeny derived from the cross of Col*^RPP1^ R3* dwarf lines (12 and 27) with Kas-2 and Kond segregated for incompatible phenotypes in the F_2_ (**S3 Table**). Incompatible phenotypes were not detected in crosses of any of the other Col*^RPP1^* lines with Kas-2 and Kond (**S3 Table**). F_2_ populations derived from Col*^RPP1^ R3* lines with lower transgene expression (14, 21, 24 and 25) also did not display incompatibility (**S3 Table**). These results suggest that individual members of the *RPP1*-like *Ler* locus, including *R3* under native expression conditions, are insufficient to condition HI with Kas-2 and Kond.

### At least two homozygous copies of *RPP1*-like L*er* genes are required for incompatibility

**Figure 4:**
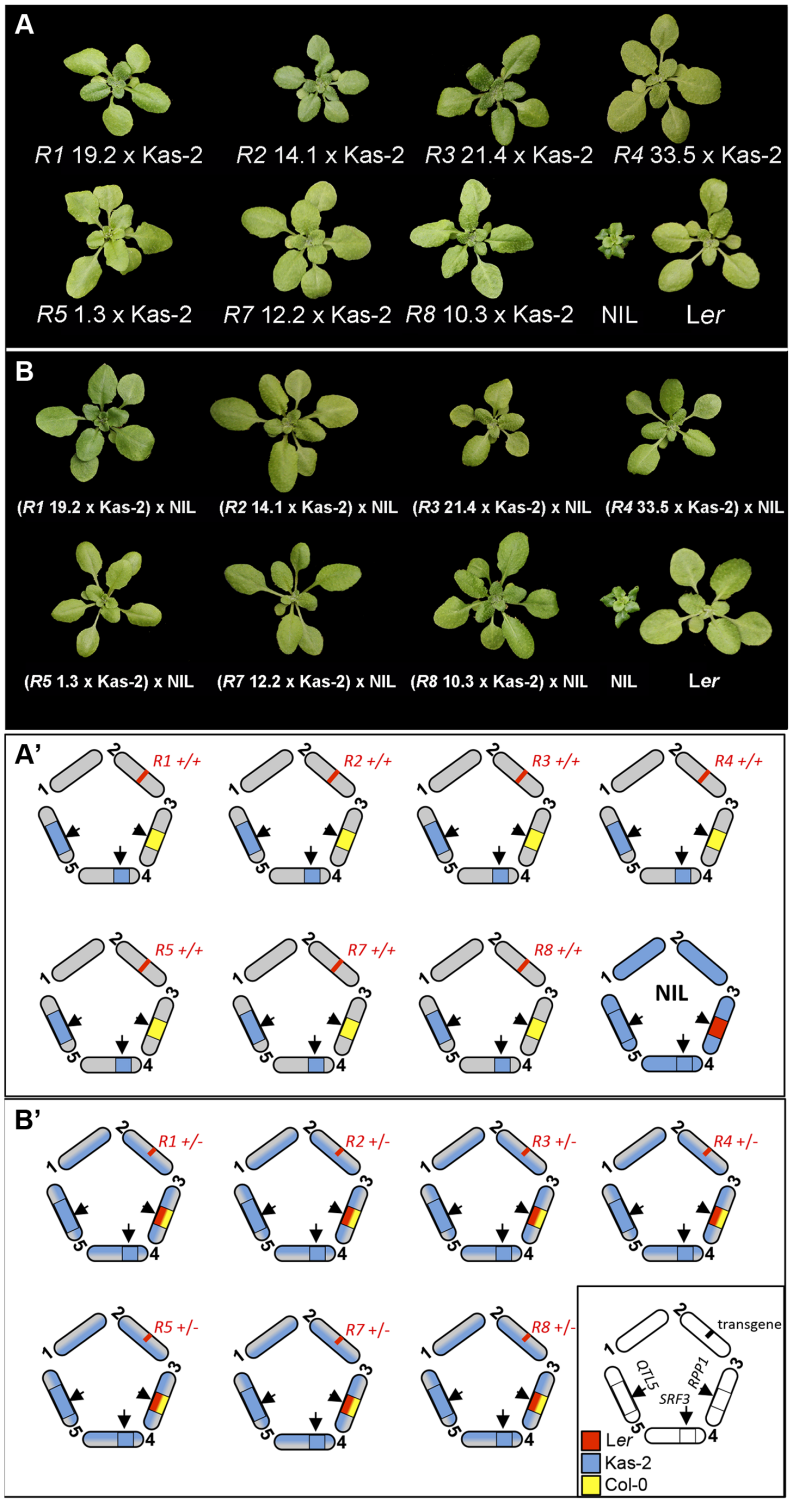
Growth phenotype at 14–16°C of Col*^RPP1^*/Kas-2 plants and *RPP1*-like L*er* hemizygous lines. (**A**) Growth phenotype of representative F_3_ plants derived from the cross of Col*^RPP1^ R1*, *R2*, *R3*, *R4*, *R5*, *R7* and *R8* transgenic lines with Kas-2, which carry their respective transgenes in combination with incompatible alleles at interacting loci. (**B**) Growth phenotype of the F_1_ progeny derived from the cross of genotypes in (**A**) with the incompatible L*er*/Kas-2 NIL [31]. Genotypes are shown in the lower panel (**A′ and B′**).

We isolated F_2_ individuals carrying each of the *RPP1*-like L*er* transgenes and Kas-2 incompatible allele combinations at *SRF3* and QTL5 (**Fig. 4A**). These lines did not exhibit HI phenotypes at 14–16°C. We then studied the effect of adding one copy of the entire *RPP1*-like L*er* cluster to these lines, maintaining homozygous Kas-2 incompatible alleles at *SRF3* and QTL5 (**Fig. 4B**), or compatible heterozygous alleles at *SRF3* (**S8 Figure**). These materials were generated by crossing the above F_2_ plants and their controls with the NIL (see Material and Methods). Growing the *RPP1*-like L*er* hemizygous lines at 14–16°C did not reveal incompatibility (**Fig. 4B**). Similar growth phenotypes to the respective controls carrying compatible heterozygous *SRF3* alleles were observed (**S8 Figure**). We concluded that immune-related HI between L*er* and Kas-2 requires a minimum expression threshold of two or more *RPP1*-like L*er* genes in combination with the Kas-2 allelic forms.

### Distribution of the *RPP1*-like L*er* haplotype in an *Arabidopsis* local population

We reported that the *RPP1*-like L*er* incompatible haplotype is rare in Europe based on analysis of thirty F_2_ populations derived from a cross of Kas-2 with different Central European accessions [23]. This contrasts with the high frequency of *SRF3* incompatible alleles found in Central Asia [23]. We also reported that the Landsberg *ERECTA* (La-0) accession is incompatible with Kas-2, indicating that the incompatible *RPP1*-like locus was already present in the parental Landsberg La-0 background [31]. Through an optimized PCR screen based on amplification of full-length *RPP1*-like L*er R1-R8* genes, we searched for the presence of a conserved *RPP1*-like L*er* haplotype in 346 *A. thaliana* accessions representing a diverse global sample (**S4 Table**). None of these shared the *RPP1*-like haplotype with L*er*. Absence of a broad distribution led us to hypothesize that the *RPP1*-like L*er* haplotype, if present in the wild, is geographically restricted.

**Figure 5:**
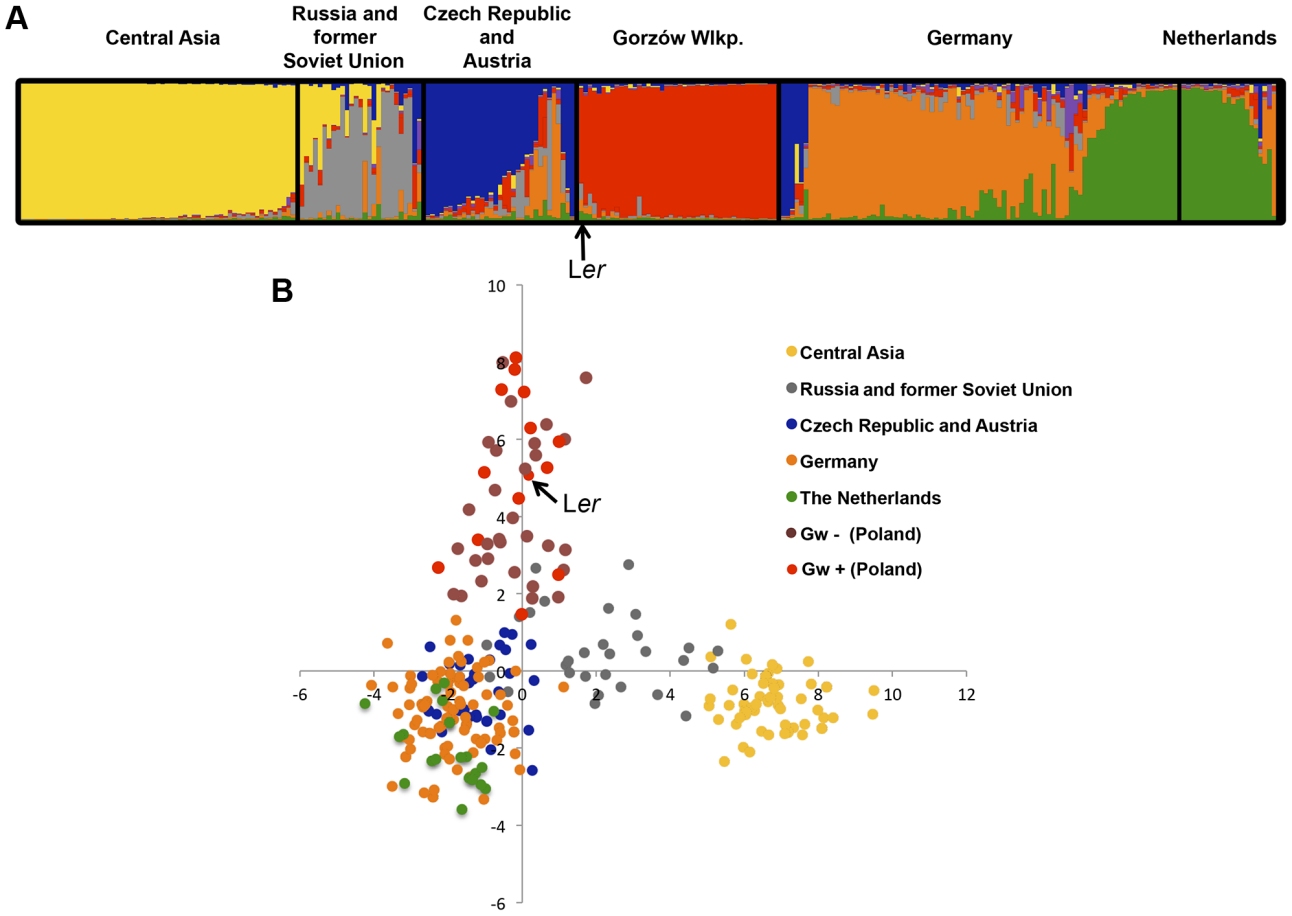
Gorzów population structure. Estimated population structure (**A**) and principal component analysis, PCA (**B**), derived from the analysis of 149 genome-wide SNP in genetically distinct individuals in the Gorzów population (n = 44) and accessions from neighboring countries (Czech Republic and Austria, n = 33; Germany, n = 88) or more distant regions (Netherlands, n = 21; Russia and former Soviet Union, n = 27; Central Asia, n = 61). Each accession is colored in segments depicting individual's estimated membership fractions in six main clusters (optimal K = 6, for lower K values see **S10 Figure**). Gw individuals carrying (Gw^+^) or not (Gw^−^) the *RPP1*-like L*er* haplotype are represented in different colors in the PCA plot.

In May 2011, we traced the origin of L*er* (Landsberg an der Warthe, Germany 1939) [37] to the area of Gorzów Wielkopolski (Poland) (**S9 Figure**). There we collected 167 individuals (named Gorzów, Gw), which were genotyped using 149 genome-wide SNPs [38]. With these markers, we could distinguish at least 44 different multi-locus haplotypes (**Figs. 5** **and** **6A**) which shared 58–72% SNPs with L*er*. This is well above the mode of ∼44% seen for arbitrary pairs of worldwide accessions [39]. Three Gw individuals (1.8% of the entire sample) carried heterozygous alleles at various markers across the genome including the *RPP1*-like cluster, suggesting that outcrossing occurs between local accessions, as previously observed [40]. Structure [41] and PCA analyses of Gw individuals and other accessions originally from neighboring countries (Austria, Czech Republic and Germany), as well as more geographically distant accessions (The Netherlands, Russia and former Soviet Union, and Central Asia), confirmed that the Gw population is most closely related to other Central European *A. thaliana* accessions and that, with K = 3 and above, forms a distinct group (**S10 Figure**).

**Figure 6:**
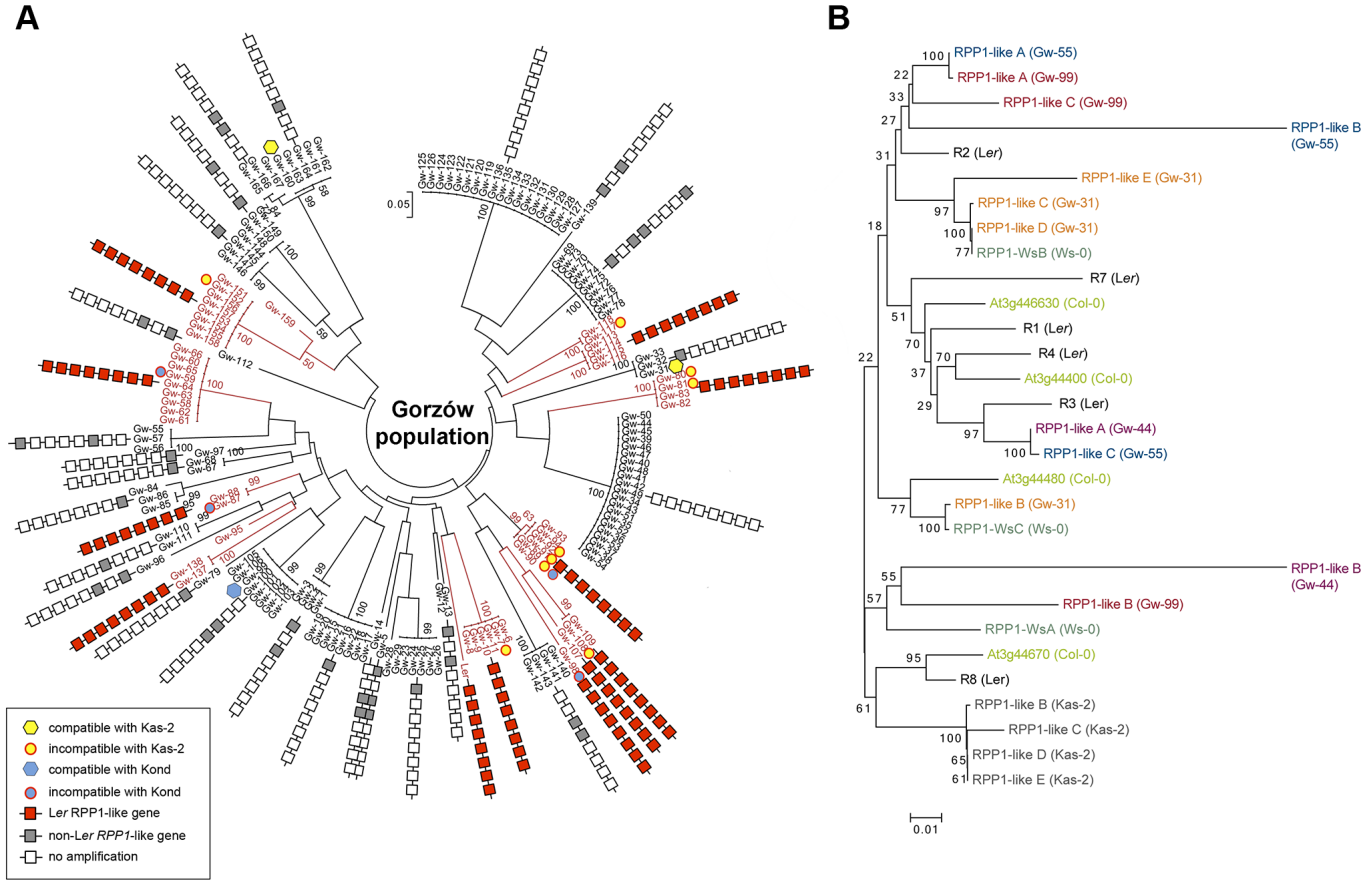
Genetic diversity of the *RPP1*-like locus in the Gorzów population. (**A**) Neighbor-joining tree showing the genome-wide genetic diversity among Gw accessions, estimated from a set of 149 genome-wide SNPs. Boxes represent genes within the *RPP1*-like haplotype (*R1* to *R8*, with *R8* the closest to the tree) conserved with L*er* (red) differing from L*er* (gray), or absent (white), based on amplification and sequencing of *RPP1*-like genes with specific primers designed for the *RPP1*-like L*er* cluster. Accessions carrying the *RPP1*-like L*er* haplotype are highlighted in red. Accessions used for crosses with Kas-2 or Kond and their compatibility/incompatibility outcome are indicated. (**B**) Neighbor-joining tree of *RPP1*-like genes in Gw^−^ and Kas-2 showing extensive allelic variation. Phylogeny is based in sequencing *RPP1*-like genes in Gw^−^ individuals (Gw-31, Gw-44, Gw-55 and Gw-99) and Kas-2.

The L*er* accession derived from plants collected in the same region in 1939 showed a close genetic relationship with the modern Gorzów population (**Fig. 5** **and S10 Figure**). About a third of Gw individuals, representing 14 different multilocus-haplotypes, shared the *RPP1*-like incompatibility genes found in L*er* (**Fig. 5B** **and S11 Figure**). This finding suggests that the derived *RPP1*-like L*er* haplotype has been maintained in the original location over at least 72 generations (assuming a generation time of one year) since the first sampling of the population in 1939 [37]. However, the *RPP1*-like L*er* haplotype has not spread in Central Europe (**S4 Table**).

For simplicity, we will refer to the accessions with *RPP1*-like L*er* alleles as Gw^+^ and those lacking it as Gw^−^. Both types of individuals were not differentiated from each other in PCA and neighbor-joining tree analyses based on genome-wide SNP data (**Figs. 5B** **and** **6A**). We also analyzed the genetic diversity of *RPP1*-like genes within the Gw^−^ genetic groups (**Fig. 6B**). Using the same primer combinations designed to amplify *RPP1*-like L*er* genes, we observed variation in the sequence and composition of the *RPP1*-like cluster in Gw^−^ individuals (**Fig. 6A**). Due to the variable nature of the cluster, we designed primers annealing to conserved *RPP1*-like sequences for cloning *RPP1*-like genes in Gw^−^ individuals. In this way, we isolated *RPP1*-like genes from four genetically different Gw^−^ accessions sharing 61%–70% SNP with L*er* (Gw-31, Gw-44, Gw-55 and Gw-99) and from Kas-2. Phylogenetic analyses, based on sequencing *RPP1*-like genes of isolated clones, showed a high degree of *RPP1*-like gene variation between Gw^−^ accessions (**Fig. 6B**). *RPP1*-like Kas-2 genes clustered together within the same branch of the tree. However, most branches were formed by *RPP1*-like genes from different accessions without an obvious relationship (**Fig. 6B**). Neighbor-Net analysis of *RPP1*-like genes from Gw^−^ and Gw^+^ accessions produced evidence for parallelograms in the network, mostly between *RPP1*-like genes from Gw^−^ accessions, which is suggestive of recombination (S12 Figure). Notably, nucleotide diversity flanking the *RPP1*-like locus was higher in Gw^−^ than Gw^+^ haplotypes, extending from ∼−50 kb to +150 kb from the locus (**S13 Figure**). These results suggest that recombination at the *RPP1*-like locus is suppressed in Gw^+^ accessions but not in Gw^−^, in agreement with observations made during the fine-mapping of the L*er* QTL in L*er*/Kas-2 heterozygous inbred families [31]. Based on these observations, we hypothesize that the derived *RPP1*-like L*er* haplotype has increased in frequency by introgression to different genetic backgrounds in the Gw population, and that low recombination has contributed to maintaining the *RPP1*-like L*er* cluster as an extended haplotype. The increase in frequency of the derived *RPP1*-like L*er* haplotype is perhaps the result of a selective advantage, drift or complex demographic events, but less likely due to a recent bottleneck, since it was found in diverse genetic backgrounds. The co-occurrence of different *RPP1*-like allelic forms in Gw^−^ individuals would be expected for *Resistance* genes in natural populations that are maintained by negative-frequency dependent selection [14]. We conclude that there is extensive diversity of *RPP1*-like genes co-occurring in a local population.

### Incompatibility between Gw and Kas-2 or Kond accessions

**Figure 7:**
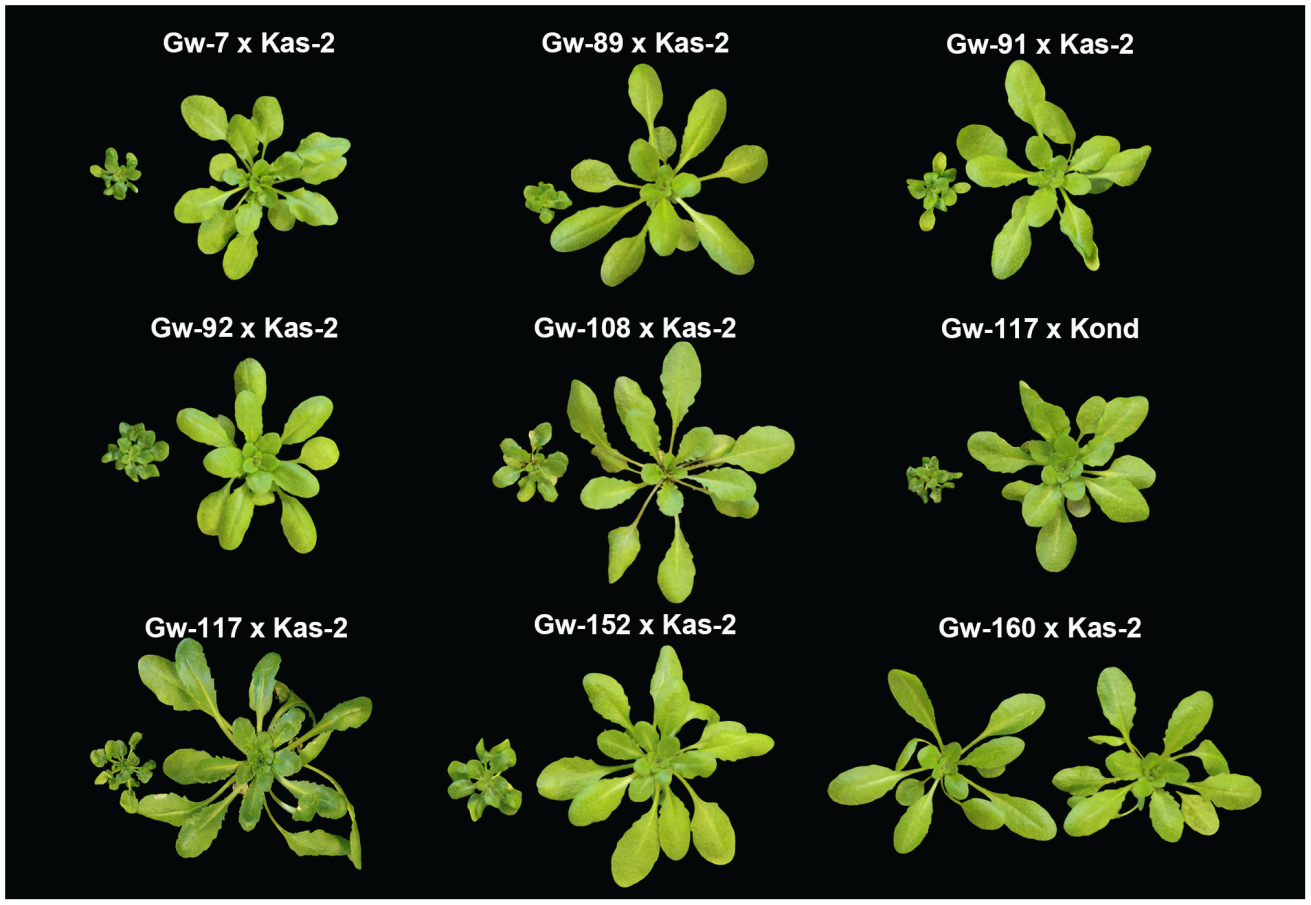
Incompatible phenotypes derived from the cross of Gw^+^/Kas-2 and Gw^+^/Kond accessions. Gw^+^ individuals were crossed to Kas-2 and Kond and their F_2_ progenies screened for the occurrence of incompatible phenotypes at 14–16°C (see **S5 Table**). Dwarf plants on the left of each cross carry homozygous Gw (L*er*) alleles at *RPP1*-like locus and homozygous *SRF3* Kas-2 or Kond alleles, which are not present in normal sized sister F_2_ plants from the same cross (right). Incompatibility is absent in the cross of Gw^−^ accessions with Kas-2 or Kond (Gw-160 as representative; see **S5 Table**).

Having found that the *RPP1*-like haplotype is maintained within genetically different individuals from a local Gorzów population, we determined whether it also conditions incompatibility with Kas-2 and Kond. Therefore 13 Gw^+^ individuals belonging to 10 mutlilocus-haplotype groups (Gw-7, Gw-59, Gw-80/81, Gw-87, Gw-89, Gw-91/92, Gw-98, Gw-108, Gw-117 and Gw-152) and three Gw^−^ controls (Gw-31, Gw-99 and Gw-160) were crossed to Kas-2 and Kond and equal numbers of F_2_ populations generated (**S5 Table**). Approximately 250 F_2_ plants per population were then scored for the segregation of incompatible phenotypes at 14–16°C. We detected the incompatibility in all crosses derived from Gw^+^ individuals with Kas-2 and Kond (**S5 Table;** **Fig. 7**). Incompatible F_2_ individuals always carried Gw (L*er*) homozygous alleles at the *RPP1*-like locus and Kas-2 or Kond homozygous alleles at *SRF3* (**S5 Table**). The data are consistent with involvement of at least two recessive loci in the Gw^+^/Kas-2 and Gw^+^/Kond incompatibilities. By contrast, F_2_ plants derived from crosses of Gw^−^ with Kas-2 did not display HI at 14–16°C (**Fig. 7** **and S5 Table**). These results show that Gw^+^ individuals carrying the *RPP1*-like L*er* haplotype are incompatible with Kas-2 and Kond. Therefore, the incompatibility reported between L*er*/Kas-2 and L*er*/Kond can be extended to a population scale.

## Discussion

The arrangement of *NLR* genes in clusters is the result of tandem, ectopic or large-scale segmental duplications that can be followed by local rearrangements. The clusters may be homogeneous, containing structurally similar *NLR* genes, or complex when formed by a heterogeneous class of *TNL* and *CNL* (*Coiled Coil-NLR*) genes [12], [42], [43]. The *RPP1*-like L*er* cluster is homogeneous and formed by a sequence of highly related *TNL* genes [31]. Different repertoires of *RPP* genes are known to recognize effectors delivered by genetically diverse strains of the *Arabidopsis* oomycete pathogen *Hpa* [44], consistent with there being coevolution between naturally evolving *Hpa* isolates and local *Arabidopsis* populations. Compared to Col-0 and *Arabidopsis lyrata* [32], accession L*er* contains six additional *RPP1*-like genes at this locus, one of which (*R6*) is a truncated form [31]. Nonreciprocal crossover is a source of copy number variation that can generate clusters of different sizes which would limit recombination [45]. Given the larger number of genes within the cluster, the *RPP1*-like L*er* haplotype appears to be a derived one. Sequence alignment and exon-intron organization suggests that *RPP1*-like genes *R3* and *R4* are the result of ectopic recombination and duplication of the *At3g44400 TNL* (**S1 Figure**). *R2* and *R8 RPP1*-like L*er* are the most closely related genes to *At3g44630* and *At3g44670* in Col-0 respectively, suggesting a common ancestry (**S1 Figure**).

Activation of defense incurs a cost for the plant, which is often translated into reduced growth and reproductive fitness [18]. However, naturally occurring alleles that lower the thresholds for activation of defense have been found at the *ACD6* locus together with wild-type alleles in *Arabidopsis* natural populations [46]. We found that the majority of *RPP1*-like L*er* genes, except *R3*, do not confer an obvious fitness cost in neutral backgrounds (Col-0) regardless of their expression levels, nor any clear advantage for disease resistance to the virulent *Hpa* isolate Noco2 (**S3, S4, S5 Figures**). *R3* overexpression in Col*^RPP1^* lines enhances resistance to *Hpa* Noco2 (**S5 Figure**) and reduces fitness at 14–16°C (**S2 Figure**), thus phenocopying L*er*/Kas-2 HI [31]. *R3* overexpression in L*er* has the same deleterious effect as in Col-0, and is therefore a background-independent phenotype. Incompatible and autoimmune phenotypes were not evident in Col*^RPP1^* lines with wild-type *R3* expression levels (**S7 Figure**). The fitness cost of *R3* overexpression suggests a need for tight *R3* transcriptional regulation to provide balanced growth and defense. Indeed, a large fraction of the methylation variability in *Arabidopsis* is detected in regions containing *NLR* genes [47], [48] and restricting *NLR* expression is important for normal plant development [49], [50].

Crossing *R3* lines exhibiting L*er* wild-type expression levels with Kas-2 and Kond failed to reconstitute HI in the F_2_ generation, nor did crossing individual *Col^RPP1^ R1*, *R2*, *R4*, *R5*, *R7* and *R8* transgenic lines with the same accessions, or adding one supporting copy of the *RPP1*-like L*er* cluster to the above lines (**Fig 4**; **S3 Table**). These results suggest that minimum expression of at least two *RPP1*-like L*er* genes is required for incompatibility with Kas-2. However, non-suppressed NIL amiRNA lines and incompatible L*er*/Kas-2 RILs exhibited higher *R3* transcript levels than suppressed NIL amiRNA lines, L*er*/Kas-2 RILs containing compatible allele combinations or incompatible RILs in an immunity suppressed *eds1-2* background (**Fig. 1B** and **Fig. 3**). Therefore, a strong positive correlation exists between *R3* expression and HI. Moreover, *R3* overexpression is sufficient to induce plant stunting (**S2 Figure**). We conclude that HI between L*er* and Kas-2 likely involves an amplification of *RPP1*-like L*er R3* expression in an *EDS1*-dependent manner. Therefore, both *RPP1*-like expression and protein differences contribute to HI. The manner in which specific Kas-2 and Kond *SRF3* allelic forms contribute to this, and a basis for co-action between *R3* and at least one other *RPP1*-like L*er* gene within the locus need to be clarified.

The multigenic nature of an incompatible locus was recently reported in an interspecific cross in rice that requires the presence of two tandem *RLK* genes for immune-related hybrid weakness [27]. Due to their close proximity and likely reduced recombination rate, genes within the *RPP1*-like L*er* cluster will tend to co-segregate, thus maintaining the locus. Underscoring this, the incompatible *RPP1*-like L*er* haplotype is maintained in the wild (Gorzów), and accessions carrying it are incompatible with Kas-2 and Kond (**Fig. 7** and **S5 Table**). An arms race based on selective sweeps of plant *R* and pathogen effector genes has been proposed to drive the coevolution between plants and pathogens. However, such dramatic ‘boom and bust’ cycles do not explain all observations made for the genetic composition and infection outcomes in wild populations [51]. Current evidence points to negative-frequency dependent selection, in which rare *R* alleles can gain a selective advantage and mitigate fitness costs, thereby promoting *R* gene cyclic dynamics and diversity [51], [52]. In addition, the possibility of neutral evolution when selection is weak, and other non-selective processes such as isolation-by-distance, might shape the genetic diversity of wild populations [53].

Here we have studied the natural distribution of the *RPP1*-like L*er* haplotype in the wild. Our screen in 346 global accessions shows that the *RPP1*-like L*er* cluster is not geographically widespread (**S4 Table**). Rather, we found it in a population of genetically related L*er* individuals in Gorzów Wielkopolski (Poland) (**Figs. 5** **and** **6**). This pattern contrasts with the wide distribution of *SRF3* Kas-2 and Kond alleles in Central Asian populations [23]. However, the genetic variation among Gorzów individuals (**Fig. 5B**) suggests that it is not a recent population. Indeed, the original La-0 and La-1 accessions were collected in 1939 [37] and the modern Gorzów population reported here in 2011. Strikingly, Gw individuals carrying the *RPP1*-like L*er* haplotype (Gw^+^) are not more genetically related to each other than to Gw^−^ individuals not carrying it (**Figs. 5B** **and** **6A**). Therefore, it is unlikely that this haplotype increased in frequency as result of a recent bottleneck. We favor an evolutionary scenario in which the derived *RPP1*-like L*er* haplotype has increased in frequency (∼30%) by introgression to different Gw genetic backgrounds (**Figs. 5B** **and** **6A**), and low recombination has helped to maintain *RPP1*-like L*er* genes in genetically different Gw^+^ individuals (**S11 and S13 Figures**).

The observed high diversity of *RPP1*-like genes in Gw^−^ individuals (**Fig. 6B**) is a pattern expected for wild populations in which multiple (rare) alleles are maintained by negative-frequency dependent selection [14], [54]. Phylogenetic network analysis of *RPP1*-like genes from Gw^+^ and Gw^−^ accessions revealed patterns suggestive of recombination between *RPP1*-like genes from Gw^−^ accessions (**S12 Figure**). Therefore, recombination between *RPP1*-like Gw^−^ genes might be a source of *RPP1*-like gene diversity in the local Gw population (**Fig. 6B**). However, such patterns were not evident between Gw^+^ and Gw^−^ *RPP1*-like genes (**S12 Figure**). This argues against the high diversity of Gw^−^ *RPP1*-like genes observed in the population being derived from multiple independent recombination/deletion events acting on the *RPP1*-like Gw^+^ (L*er*) cluster.

Increased expression of *RPP1*-L*er R3* might confer a selective advantage in disease resistance or a fitness cost in terms of growth and reproduction at moderately low temperature (14–16°C), highlighting the potential importance of trade-offs and genotype-by-environment interactions in the evolutionary dynamics of *RPP1*-like genes in wild populations. Whether the increase in frequency of the *RPP1*-L*er* haplotype in Gorzów is due to drift, demography or selection requires further investigation. Such an evolutionary study will entail determining the genetic diversity of *R* genes and local pathogen populations as well as fitness effects modulated by local environment. These complex interactions, involving also beneficial or commensal microbial communities that might compete with pathogens for resources [55], are likely to shape how plants and pathogens coevolve in nature.

## Materials and Methods

### Plant materials and growth conditions

The identity and stock numbers of the *Arabidopsis thaliana* accessions used in this study are listed in S4 Table. Seeds were obtained from the Nottingham Arabidopsis Stock Center (NASC) or collected by the authors. Seeds from the Gorzów population were harvested in Gorzów Wielkopolski (Poland) in May 2011, and amplified between June–October 2011 as single seed descent at the greenhouse facilities of the Max Planck Institute for Plant Breeding Research (Cologne, Germany). Progeny of Gw individuals was directly used for SNP genotyping (see below). The genotype of the incompatible L*er*/Kas-2 NIL used in this study has been described before [31]. For growth of plants, seeds were stratified on wet filter paper at 4°C in the dark for 2 to 4 days, and transferred to soil for germination. Plants were germinated and grown under 12 h dark/12 h light cycles, 14°C/16°C and 70% relative humidity in growth chambers (Percival Scientific, USA).

### amiRNA lines

amiRNA constructs in binary vectors targeting *RPP1*-like genes used in this study, KB209 (21mer: TGACACATAAACTCCATCGGT), KB212 (21mer: TACATTTCAACTGCGAGCGTC) and KB228 (21mer: TATATCCGTAATGATTGCGGC) designed using WMD3 [56], were transformed into L*er*/Kas-2 NIL plants by floral dip [57]. For the selection of transgenic lines, seeds were surface-sterilized and selected on MS ½ solid media containing 50 µg/ml kanamycin (Sigma-Aldrich) and 200 µg/ml ticarcillin/clavulanic acid 15∶1 (Duchefa). T_1_ plants were transferred to soil at 23°C and T_2_ homozygous lines isolated by segregation analyses of T_3_ seeds on selective media.

### Gene expression analyses

Total RNA isolated from 5-week old plants was extracted using TRIzol reagent (Invitrogen). Two micrograms of RNA was treated with DNAse I (Invitrogen) and first strand cDNA synthesized using Superscript II (Invitrogen) and oligo dT. Quantitative real-time PCR using SYBR Green I dye method was performed on Roche LightCycler 480 II detector system following the PCR conditions: 95°C 2 min, 40 cycles (95°C, 15 s; 60°C, 30 s; 68°C, 20 s). Standard curves were performed for quantification. Primers used for gene expression analyses are listed in S2 Table. The specificity of *RPP1*-like L*er* oligonucleotides was determined by comparison of the amplification efficiency using a series of equimolecular premixes of *RPP1*-like L*er R1*-*R8* plasmid DNAs in which the target genes were absent or present. qRT-PCR analyses were always performed on at least three biological replicates with three technical replicates each using *UBQ10* (*At4g05320*) as housekeeping gene.

### Generation of Col-0 *^RPP1^* ^-like L*er*^ transgenic lines

Genomic regions of *RPP1*-like L*er* genes were amplified from 200 ng of freshly extracted genomic DNA (BioSprint Workstation, Qiagen) of the L*er* accession (code number N20), using primer combinations listed in Table S2 and LA Taq DNA polymerase (Takara). PCR conditions were: 94°C 5 min, followed by 30 cycles (94°C, 15 s; 55°C, 30 s; 68°C, 4 min), 68°C 10 min. PCR products were separated on 1% agarose gel stained with ethidium bromide, and purified by gel scission (Gel extraction Kit, Qiagen). Purified fragments were cloned into pGEM T-easy (Promega), and the clones sequenced using T7, SP6 and primers listed in Table S2. Sanger sequencing was performed at the Max Planck Genome Center Cologne (Cologne, Germany). The *RPP1*-like L*er* genes were released from pGEMT-easy by digestion with Not I and cloned into the pCambia1300 binary vector (www.cambia.org) modified to contain the PspOMI site in the MCS. The resulting clones were transformed into *Agrobacterium tumefaciens* GV3101 pMP90 strain [58] for transformation of Col-0 plants [57]. T_0_ seeds were selected on MS ½ media supplemented with hygromycin 15 µg/ml and 200 µg/ml ticarcillin/clavulanic acid 15∶1 (Duchefa). Homozygous lines with single T-DNA insertions were determined by segregation analyses and selected for further analyses.

### Generation of *RPP1*-like L*er* hemizygous lines

To generate *RPP1*-like hemizygous lines, we isolated multiple F_2_ plants from populations described in Table S3 with the genotypes: *RPP1*-like L*er* transgene (*+/+*), *RPP1*-like locus (*Col-0/Col-0*), *SRF3*: *Kas-2/Kas-2* or *Col-0/Col-0*, QTL5: *Kas-2/Kas-2*. F_3_ plants were then crossed to the NIL (*RPP1-like*: L*er*/L*er*; *SRF3*: Kas-2/Kas-2; QTL5: Kas-2/Kas-2) and isolated F_1_ plants carrying one copy of *RPP1*-like L*er* transgene (*+/−*), the *RPP1*-like L*er* cluster in hemizygosity (L*er*/Col-0) and incompatible (*Kas-2/Kas-2*) or compatible (*Kas-2/Col-0*) alleles at *SRF3*, and *Kas-2/Kas-2* at QTL5, as confirmed by genotyping using markers previously reported [23], [31].

### Histochemical analyses and pathogen infection assays

Cell death was determined by staining with lactophenol trypan blue [31] and visualization under light microscope (Axioplan, Carl Zeiss). Images were captured in a Leica DFC490 digital camera. Infection with *Hpa* Cala2 and Noco2 isolates was performed as described [23], [31]. Plant cell death and *Hpa* infection structures were visualized under light microscope after 4 days of infection.

### Screen of *RPP1*-like L*er* haplotype in *Arabidopsis* accessions

Genomic DNA from 5-week-old *Arabidopsis* accessions listed in Table S4 was extracted using DNA BioSprint Workstation (Qiagen) and arrayed in 96-well plates including L*er* controls on every plate. 200 ng of freshly extracted DNA was used for amplification of full-length *RPP1*-like *R1-R8* L*er* genes using primer combinations listed in Table S2 and LA Taq DNA polymerase (Takara). Amplification of *UBQ10* was used as control. PCR conditions were as described above for cloning *RPP1*-like L*er* genes. Products of the PCR reaction were separated on 1% agarose gels stained with EthBr. Amplicons and sizes were documented using the Gel Doc XR (BioRad) system.

### Genotyping, structure, PCA and phylogenetic analyses

Genomic DNA from Gw accessions was extracted from leaves of 5-week old plants grown in the greenhouse using the BioSprint Workstation (Qiagen) platform by following manufacturer's instructions. SNP multilocus genotypes [38] were determined using the genotyping facility services of the University of Chicago (Chicago, USA). Presence/absence of *RPP1*-like L*er* haplotype in the Gw population was determined by PCR amplification and sequencing of *RPP1*-like genes using specific primers (Table S2). The population structure of the Gw population was inferred using the software STRUCTURE [41] and previously described settings [23]. To adjudicate the correct number of genetic clusters K, we applied the Δ*K* method [59] in combination with the absolute value of ln P(X|K). Principal component analysis was performed using R. Neighbor joining-tree was performed from aligned sequences using Mega6.06 and 5000 bootstrap repetitions. Neighbor-net was constructed using SplitsTree4 [60]. Nucleotide diversity was computed using DnaSP (DNA Sequence Polymorphism version 5.10) [61].

### Cloning of *RPP1*-like genes in Gw^-^ accessions

Genomic DNA was extracted from leaves of Gw accessions using DNeasy Plant Mini Kit (Qiagen) according to manufacturer's instructions. *RPP1*-like genes were amplified by PCR using RA+RC and RB+RC primer combinations and Phusion High-Fidelity DNA Polymerase (Thermo Scientific) and the same PCR conditions as above (**Table S2**). Amplified fragments were purified by gel scission (Gel Extraction Kit, Qiagen), ligated into the vector pSPARKII (Canvax) and transformed into *E. coli* DH10B (Clontech). The plasmid DNA from at least 16 independent clones was extracted using Plasmid MiniPrep Kit (Qiagen) and used for Sanger sequencing (**Table S2**) to identify unique clones. Sequences obtained were assembled using MacVector 12.7.5 and single contigs aligned to the *A. thaliana* genome using BLAST (http://blast.ncbi.nlm.nih.gov/Blast.cgi) to confirm their identity based on similarity to known *RPP1* and *RPP1*-like genes. Sequences are deposited in GenBank under accession numbers KM575915–KM575930.

## Supporting Information

S1 Figure Neighbor-joining tree of *RPP1*-like genes and schematic exon-intron organization. *RPP1*-like phylogeny was determined using gene sequences of *RPP1*-like L*er* members (accession number FJ446580) and *RPP1*-like Col-0 genes (*At3g44400*, *RPP1*:*At3g44480*, *At3g44630* and *At3g44670*). Exons are represented by gray boxes and introns by horizontal lines. Leucine-rich repeats, predicted N-myristoylation sites and stop codons are indicated.
(TIF)

S2 Figure Temperature-dependent incompatible phenotype of Col*^RPP1^ R3* over expressor lines. Growth phenotype of 5-week old Col*^RPP1^ R3* over expressor line 12.1 grown at 14–16°C (left) or 20–22°C (right).
(TIF)

S3 Figure Growth phenotype of Col*^RPP1^* lines. 7-week old Col*^RPP1^ R1*, *R2*, *R4*, *R5*, *R7* and *R8* lines with high *RPP1*-like transgene expression and *R3* line with wild-type (L*er*) transgene expression levels grown at 14–16°C.
(TIF)

S4 Figure Cell death phenotypes of Col*^RPP1^* lines. Microscopic examination of cell death (red arrows) revealed by trypan blue staining of 5-week old Col*^RPP1^* lines grown at 14–16°C. *sid2-1*, *isochorismate synthase* mutant. Scale bar, 500 µm.
(TIF)

S5 Figure Disease resistance phenotypes of Col*^RPP1^* lines to *H. arabidopsidis* Noco2. Two week old Col*^RPP1^* lines grown at 14–16°C were inoculated with the virulent *Hpa* isolate Noco2. Cell death (red arrows) and growth of the pathogen mycelium (orange arrows) was observed by trypan blue staining and microscopic examination 4 days postinoculation. *sid2-1*, *isochorismate synthase* mutant. Scale bar, 500 µm.
(TIF)

S6 Figure Hypersensitive response (HR) phenotypes of Col*^RPP1^* lines to *H. arabidopsidis* Cala2. The same lines and growth conditions in S5 Figure were used for inoculation with the avirulent *Hpa* isolate Cala2. Cell death (red arrows) was observed by trypan blue staining and microscopic examination 4 days postinoculation. Scale bar, 500 µm.
(TIF)

S7 Figure Cell death and disease resistance phenotypes of Col*^RPP1^ R3* (line 14.1) to *H. arabidopsidis* Noco2 and Cala2 isolates. Cell death (red arrows) and growth of the pathogen mycelium (orange arrows) was observed by trypan blue staining and microscopic examination four days postinoculation. Growth and inoculation conditions were performed as in S5 and S6 Figures. Scale bar, 500 µm.
(TIF)

S8 Figure Growth phenotype at 14–16°C of *RPP1*-like L*er* hemizygous lines which carry compatible (heterozygous) alleles at *SRF3*. Genotypes of the lines are shown below.
(TIF)

S9 Figure Collection sites of *Arabidopsis* individuals in Gorzów Wlkp. Circles represent the collection sites of unique genotypes in the Gorzów population. The fraction (%) of SNP shared with/differing from L*er* is shown in blue/red. Individuals carrying the *RPP1-*like L*er* haplotype are circled in yellow. GPS positions of collection sites are indicated.
(TIF)

S10 Figure Gorzów population structure analyses at lower K values. Population structure of Gorzów and other accessions from neighboring countries (Czech Republic, Austria and Germany) and from more distant regions (Netherlands, Russia and former Soviet Union, Central Asia) determined at K = 2 to K = 5.
(TIF)

S11 Figure Neighbor-joining tree of *RPP1*-like genes in Gw^+^. Phylogeny is based in sequencing the polymorphic *LRR* domain except *R6* (*TIR*) of *RPP1*-like genes in 12 Gw^+^ individuals: Gw-7, Gw-59, Gw-81, Gw-87, Gw-89, Gw-91, Gw-95, Gw-98, Gw-108, Gw-117, Gw-152 and Gw-159.
(TIF)

S12 Figure Phylogenetic analysis of *RPP1*-like genes. Neighbor-net representation of *RPP1*-like genes in Gw^−^ (blue), Gw^+^/L*er* (red), Col-0 (*At3g44400*, *At3g44480*, *At3g44630* and *At3g44670*) and Ws-0 (*RPP1-WsA*, *RPP1-WsB* and *RPP1-WsC*) (black). Parallelograms in the network may indicate recombination events.
(TIF)

S13 Figure Nucleotide diversity across the *RPP1*-like locus in Gw^+^ and Gw^−^. The data were obtained from sequencing flanking genes in Gw^+^ (Gw-7, Gw-59, Gw-81, Gw-87, Gw-89, Gw-91, Gw-95, Gw-98, Gw-108, Gw-117, Gw-152 and Gw-159) and Gw^−^ (Gw-2, Gw-19, Gw-23, Gw-31, Gw-44, Gw-55, Gw-69, Gw-99, Gw-119, Gw-140, Gw-144 and Gw-160) accessions at indicated intervals. Lower nucleotide diversity in Gw^+^ compared to Gw^−^ accessions suggests that recombination is suppressed in accessions carrying the *RPP1*-like L*er* cluster (Gw^+^).
(TIF)

S1 Table Sequences of amiRNAs and predicted complementarity.
(DOCX)

S2 Table List and sequences of oligonucleotides.
(DOCX)

S3 Table Segregation analyses for the occurrence of incompatible phenotypes at 14–16°C in F_2_ populations derived from the cross of Col*^RPP1^* lines to Kas-2 and Kond. Color scale represents the variation in transgene expression observed between lines.
(DOCX)

S4 Table List of *Arabidospsis thaliana* accessions used in this study and presence (+) or absence (−) of *RPP1*-like L*er* haplotype.
(DOCX)

S5 Table Segregation analyses for the occurrence of incompatible phenotypes at 14–16°C in F_2_ populations derived from the cross of Gw^+^ and Gw^−^ accessions to Kas-2 and Kond.
(DOCX)
