# Supplementary figures and images for: Analysis of a Plant Complex Resistance Gene Locus Underlying Immune-Related Hybrid Incompatibility and Its Occurrence in Nature
Source: PLoS Genet. 2014 Dec 11;10(12):e1004848. doi: 10.1371/journal.pgen.1004848 (PMC4263378; doi:10.1371/journal.pgen.1004848)

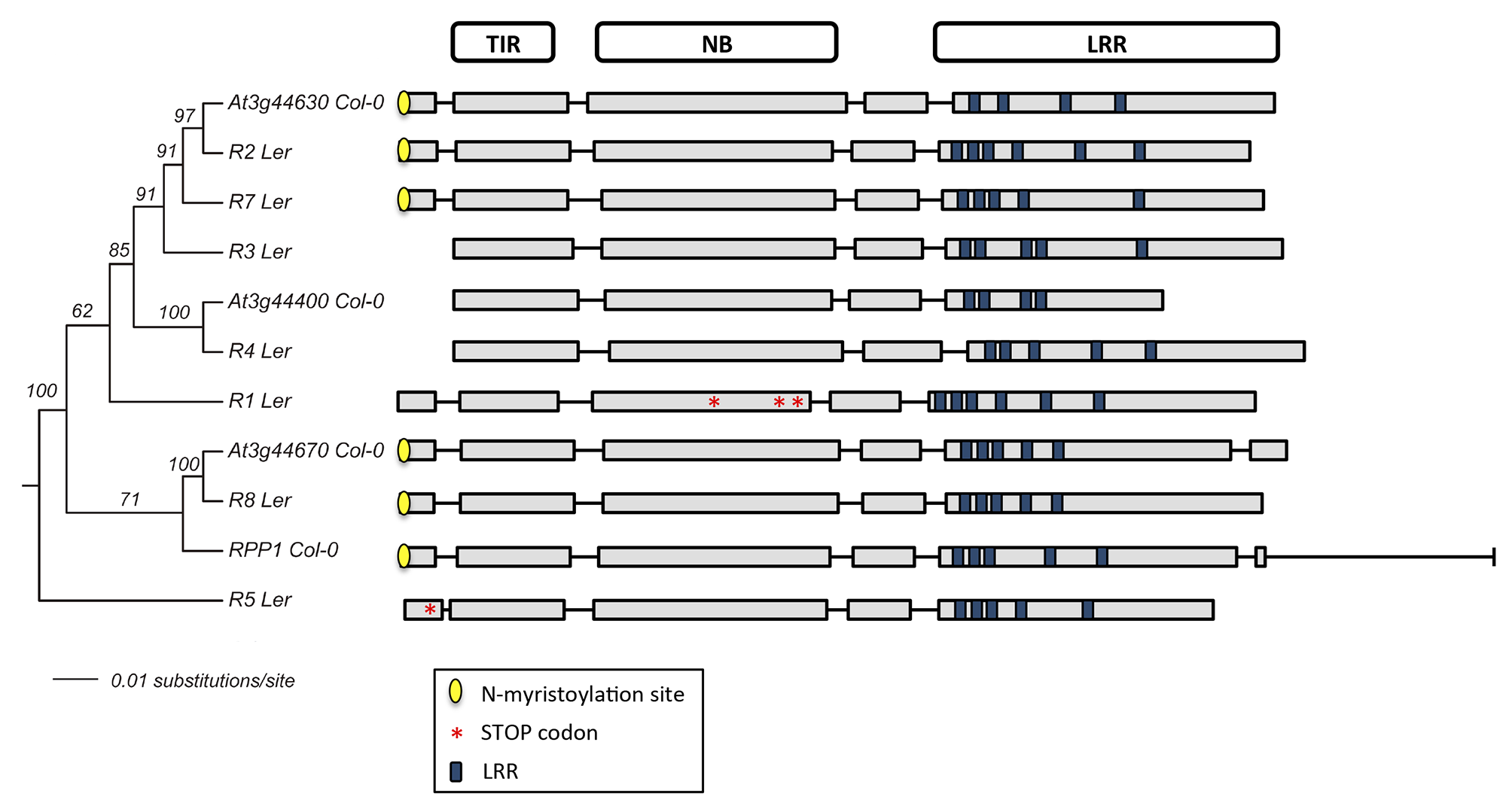

Supplement: S1 Figure — Neighbor-joining tree of RPP1-like genes and schematic exon-intron organization. RPP1-like phylogeny was determined using gene sequences of RPP1-like Ler members (accession number FJ446580) and RPP1-like Col-0 genes (At3g44400, RPP1:At3g44480, At3g44630 and At3g44670). Exons are represented by gray boxes and introns by horizontal lines. Leucine-rich repeats, predicted N-myristoylation sites and stop codons are indicated. (TIF) [file pgen.1004848.s001.tif]

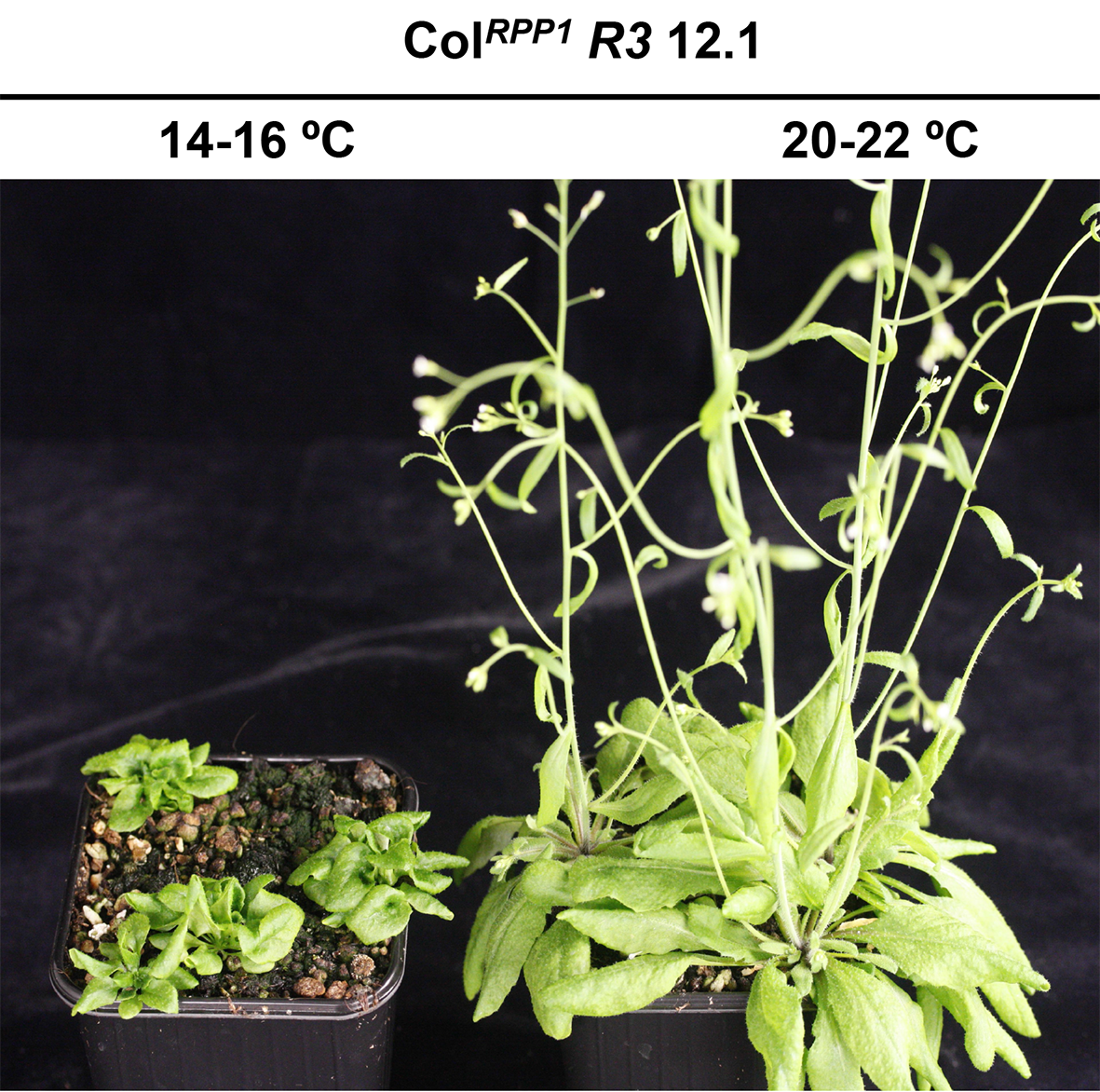

Supplement: S2 Figure — Temperature-dependent incompatible phenotype of ColRPP1 R3 over expressor lines. Growth phenotype of 5-week old ColRPP1 R3 over expressor line 12.1 grown at 14–16°C (left) or 20–22°C (right). (TIF) [file pgen.1004848.s002.tif]

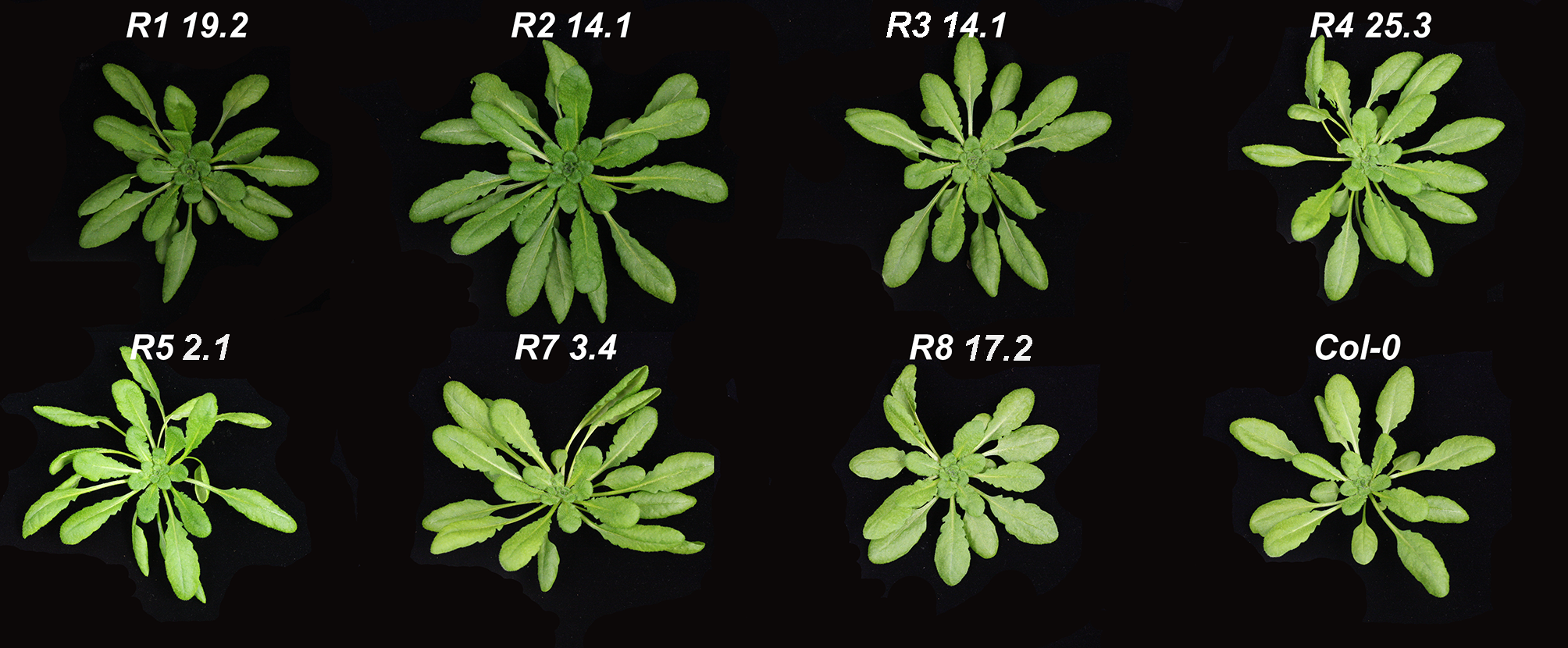

Supplement: S3 Figure — Growth phenotype of ColRPP1 lines. 7-week old ColRPP1 R1, R2, R4, R5, R7 and R8 lines with high RPP1-like transgene expression and R3 line with wild-type (Ler) transgene expression levels grown at 14–16°C. (TIF) [file pgen.1004848.s003.tif]

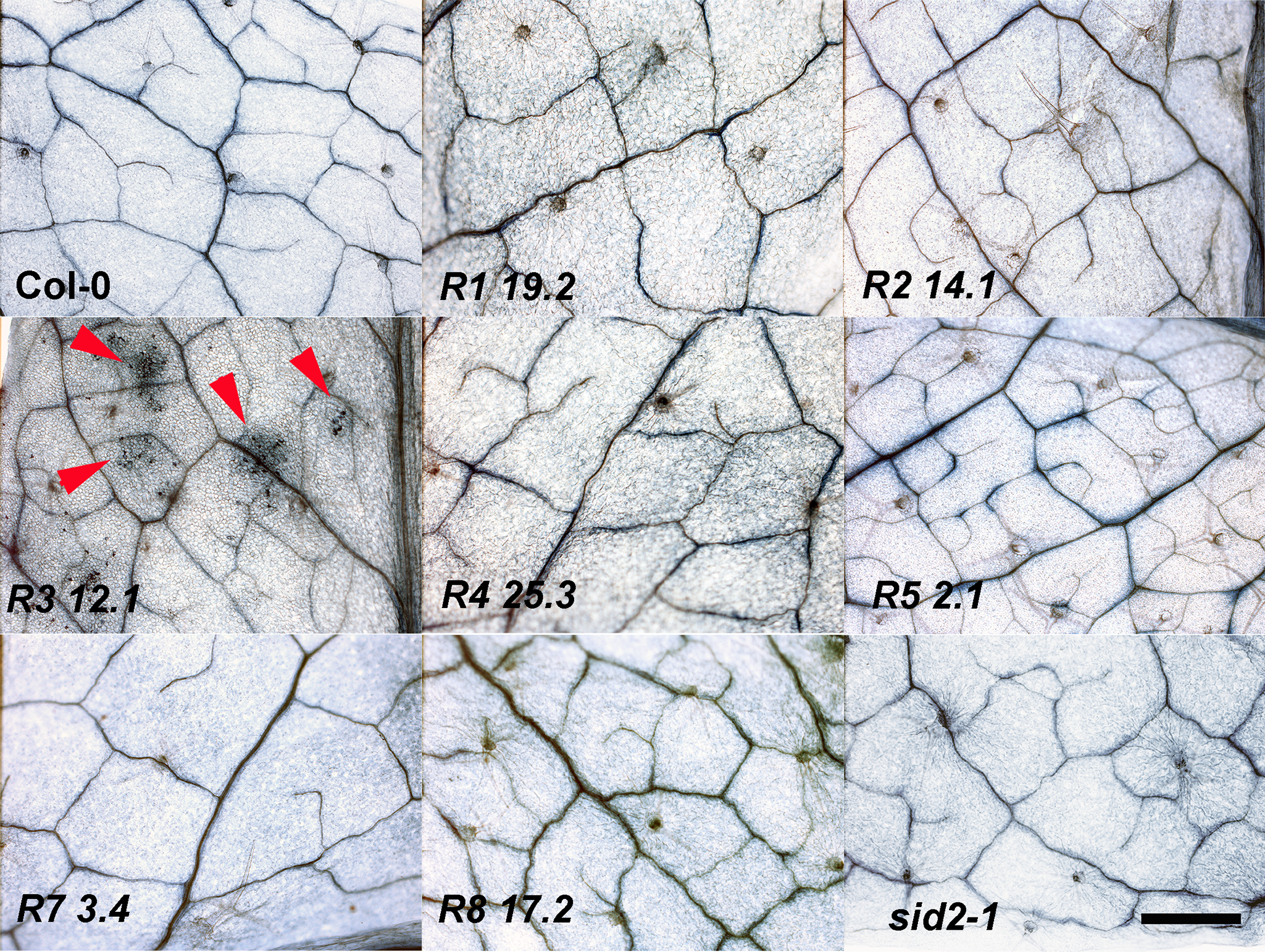

Supplement: S4 Figure — Cell death phenotypes of ColRPP1 lines. Microscopic examination of cell death (red arrows) revealed by trypan blue staining of 5-week old ColRPP1 lines grown at 14–16°C. sid2-1, isochorismate synthase mutant. Scale bar, 500 µm. (TIF) [file pgen.1004848.s004.tif]

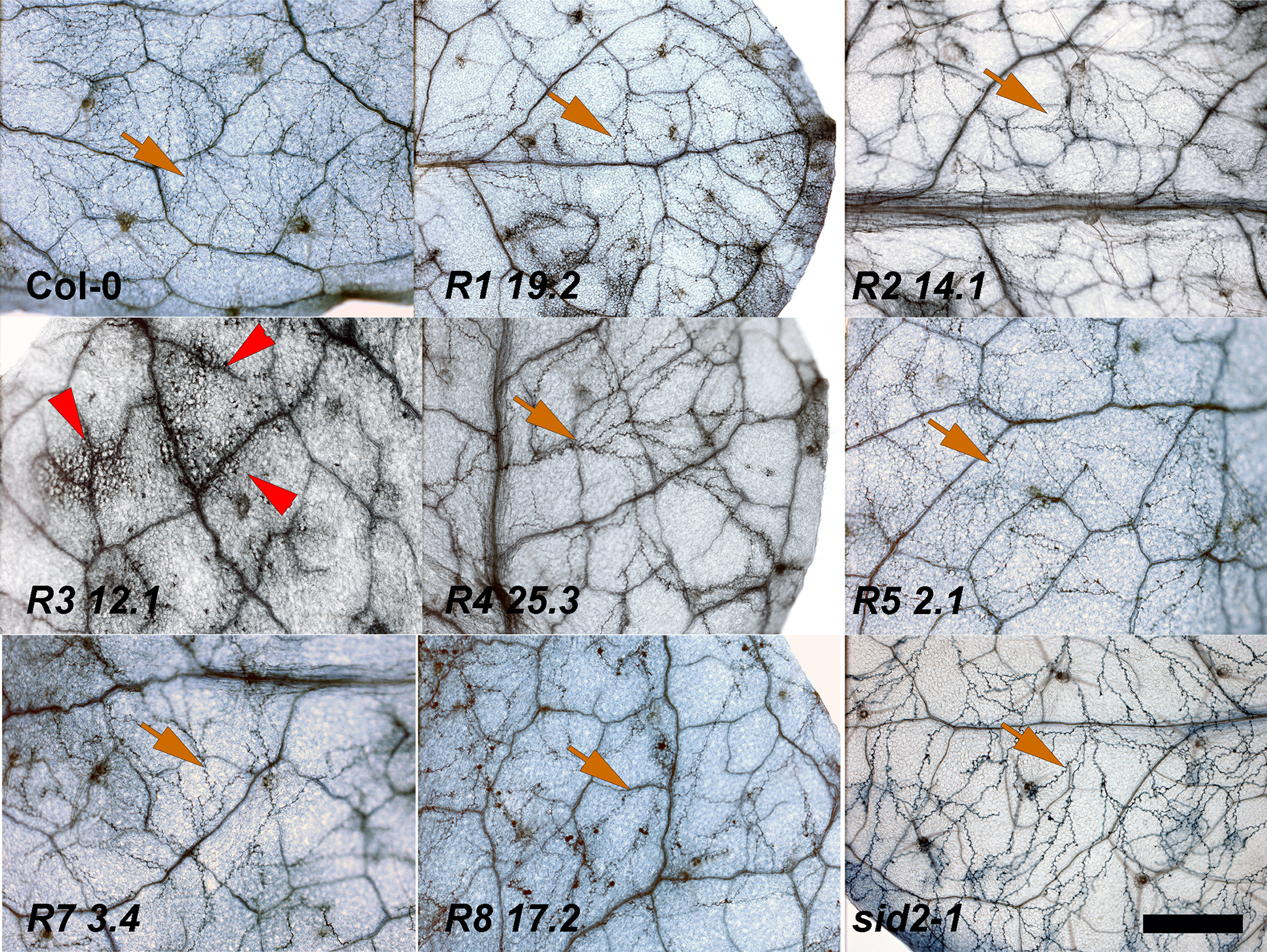

Supplement: S5 Figure — Disease resistance phenotypes of ColRPP1 lines to H. arabidopsidis Noco2. Two week old ColRPP1 lines grown at 14–16°C were inoculated with the virulent Hpa isolate Noco2. Cell death (red arrows) and growth of the pathogen mycelium (orange arrows) was observed by trypan blue staining and microscopic examination 4 days postinoculation. sid2-1, isochorismate synthase mutant. Scale bar, 500 µm. (TIF) [file pgen.1004848.s005.tif]

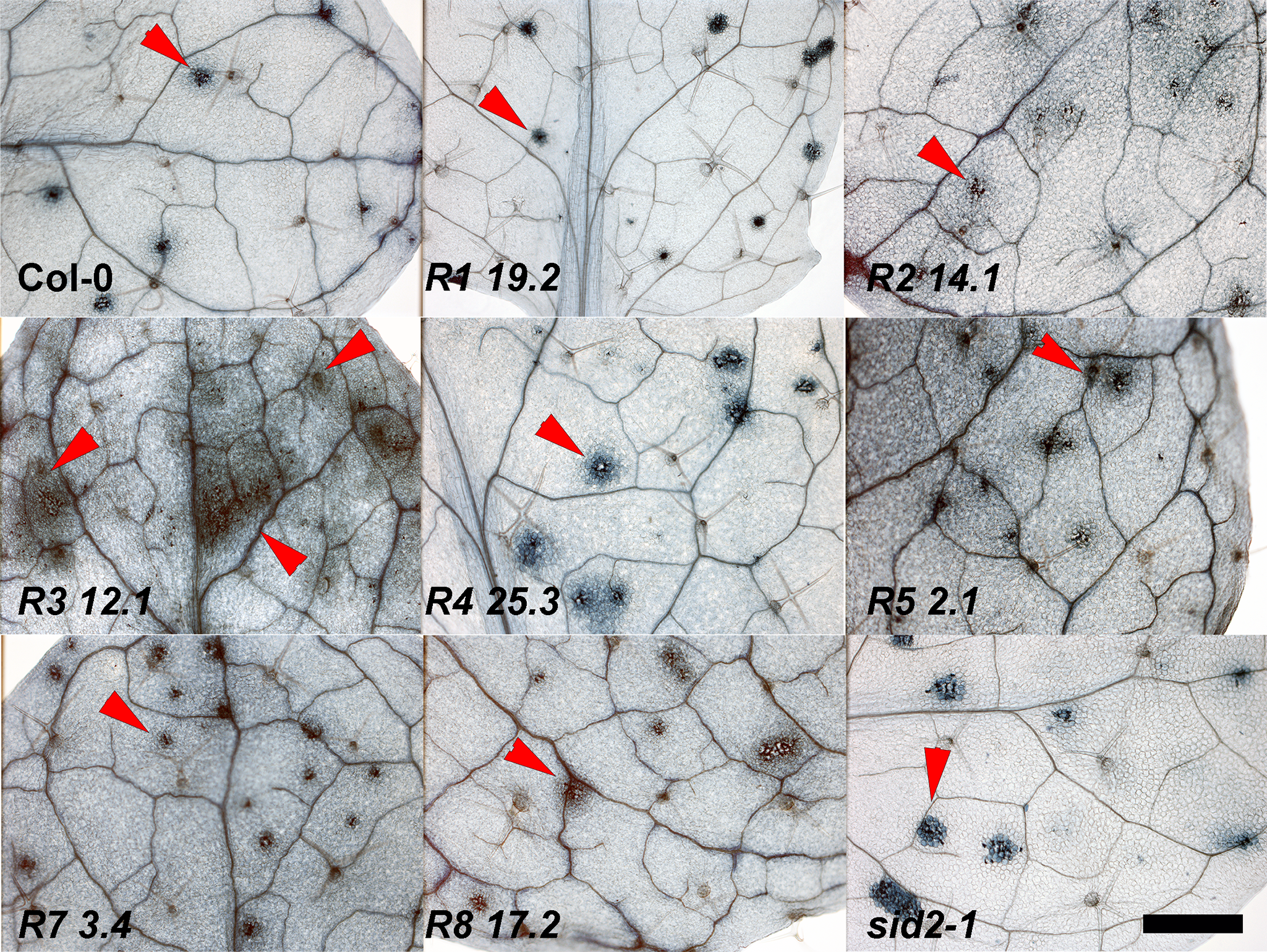

Supplement: S6 Figure — Hypersensitive response (HR) phenotypes of ColRPP1 lines to H. arabidopsidis Cala2. The same lines and growth conditions in S5 Figure were used for inoculation with the avirulent Hpa isolate Cala2. Cell death (red arrows) was observed by trypan blue staining and microscopic examination 4 days postinoculation. Scale bar, 500 µm. (TIF) [file pgen.1004848.s006.tif]

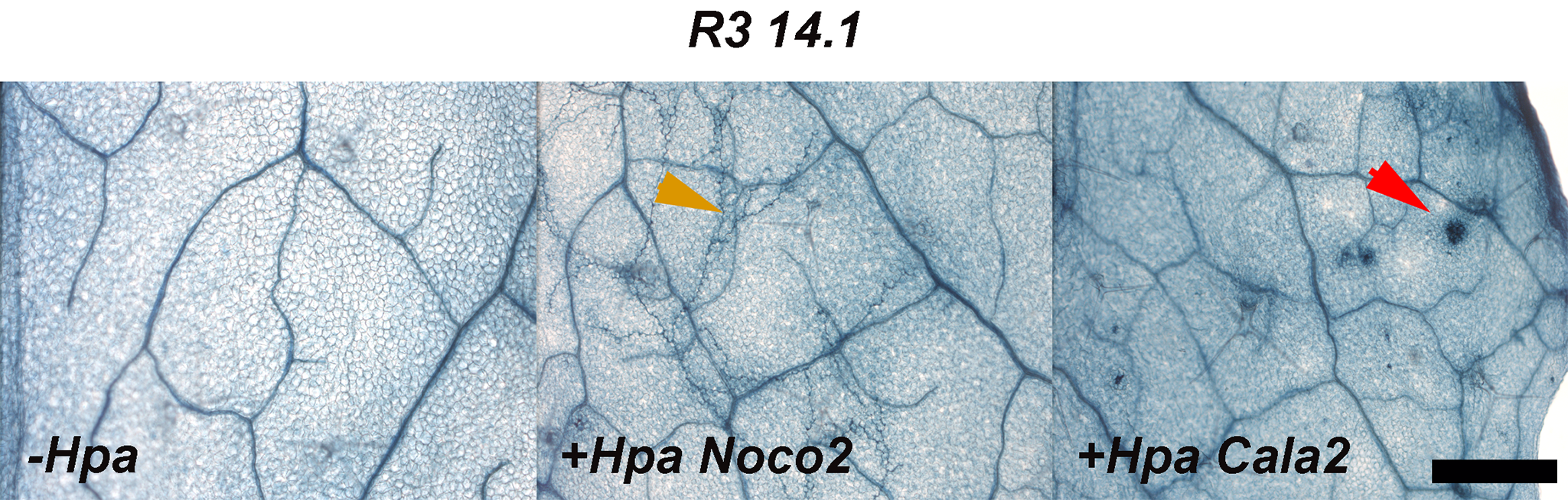

Supplement: S7 Figure — Cell death and disease resistance phenotypes of ColRPP1 R3 (line 14.1) to H. arabidopsidis Noco2 and Cala2 isolates. Cell death (red arrows) and growth of the pathogen mycelium (orange arrows) was observed by trypan blue staining and microscopic examination four days postinoculation. Growth and inoculation conditions were performed as in S5 and S6 Figures. Scale bar, 500 µm. (TIF) [file pgen.1004848.s007.tif]

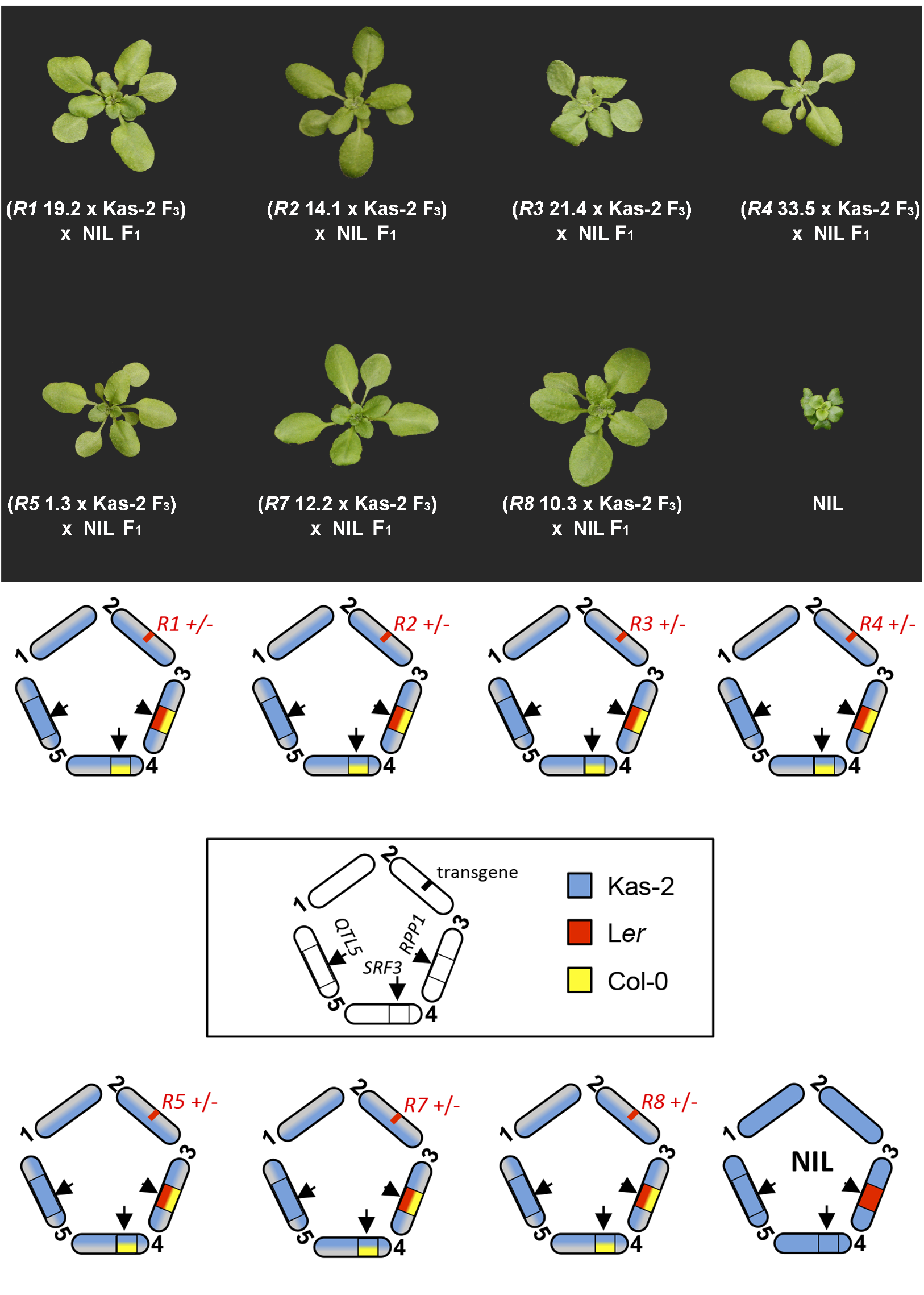

Supplement: S8 Figure — Growth phenotype at 14–16°C of RPP1-like Ler hemizygous lines which carry compatible (heterozygous) alleles at SRF3. Genotypes of the lines are shown below. (TIF) [file pgen.1004848.s008.tif]

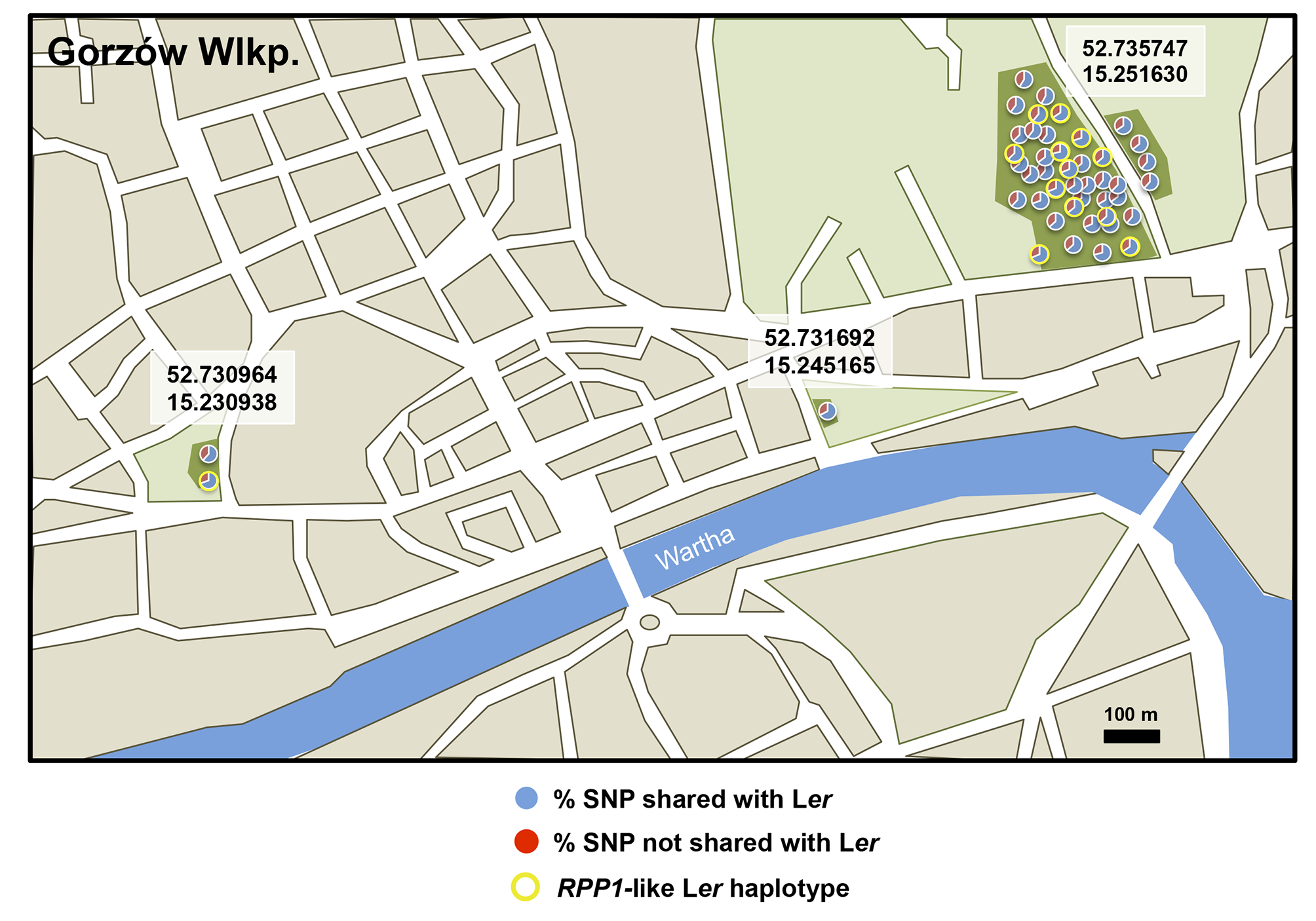

Supplement: S9 Figure — Collection sites of Arabidopsis individuals in Gorzów Wlkp. Circles represent the collection sites of unique genotypes in the Gorzów population. The fraction (%) of SNP shared with/differing from Ler is shown in blue/red. Individuals carrying the RPP1-like Ler haplotype are circled in yellow. GPS positions of collection sites are indicated. (TIF) [file pgen.1004848.s009.tif]

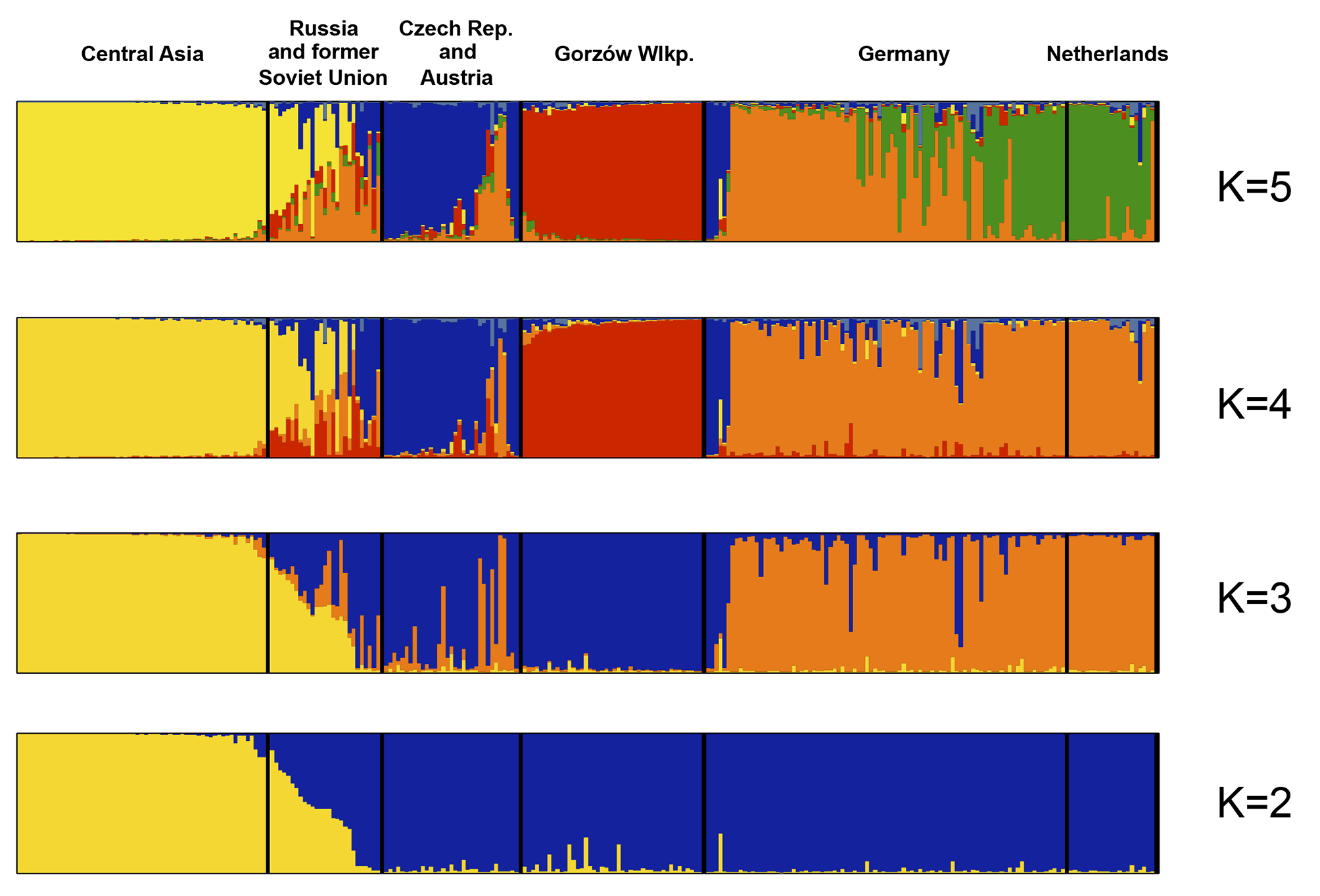

Supplement: S10 Figure — Gorzów population structure analyses at lower K values. Population structure of Gorzów and other accessions from neighboring countries (Czech Republic, Austria and Germany) and from more distant regions (Netherlands, Russia and former Soviet Union, Central Asia) determined at K = 2 to K = 5. (TIF) [file pgen.1004848.s010.tif]

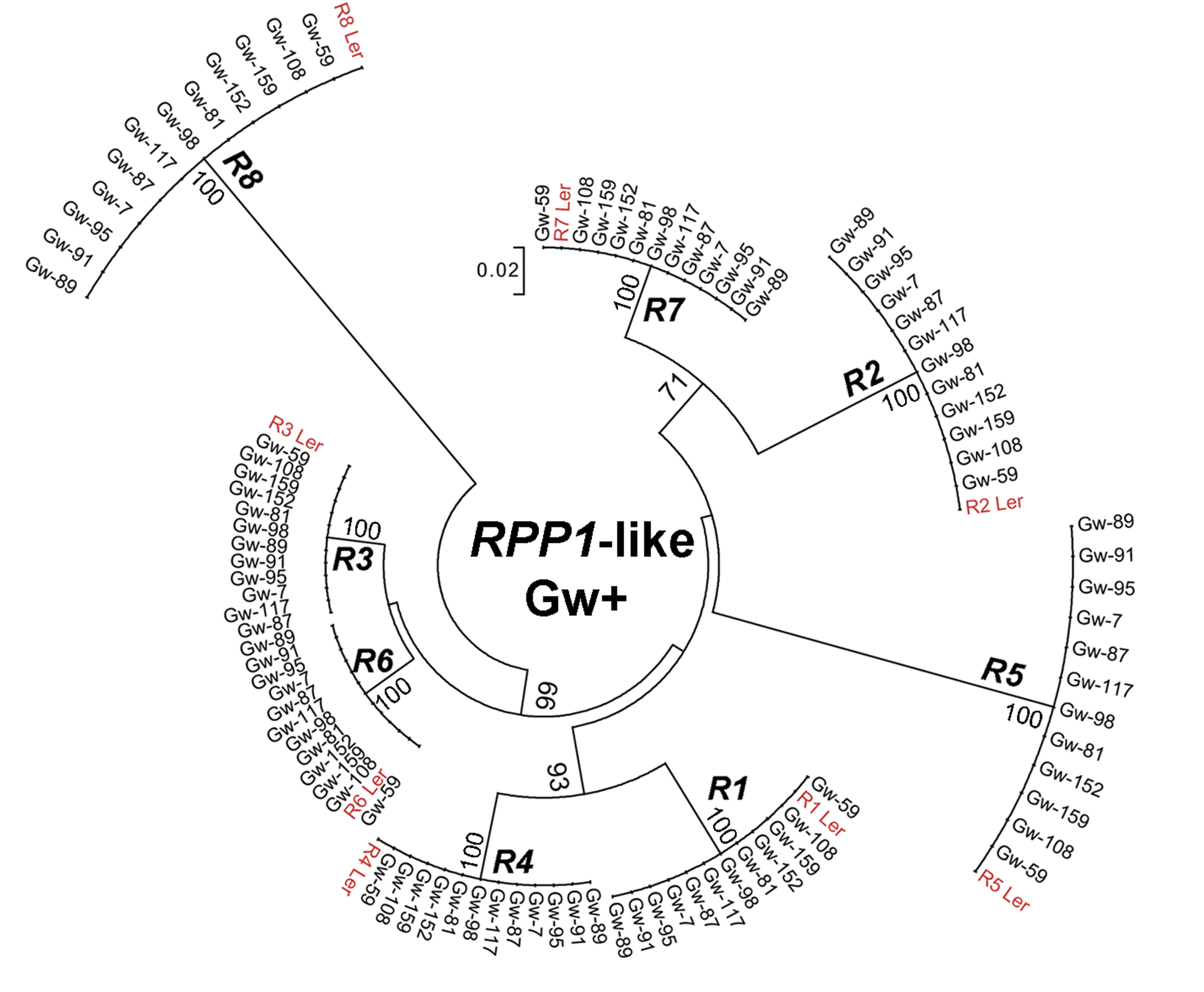

Supplement: S11 Figure — Neighbor-joining tree of RPP1-like genes in Gw+. Phylogeny is based in sequencing the polymorphic LRR domain except R6 (TIR) of RPP1-like genes in 12 Gw+ individuals: Gw-7, Gw-59, Gw-81, Gw-87, Gw-89, Gw-91, Gw-95, Gw-98, Gw-108, Gw-117, Gw-152 and Gw-159. (TIF) [file pgen.1004848.s011.tif]

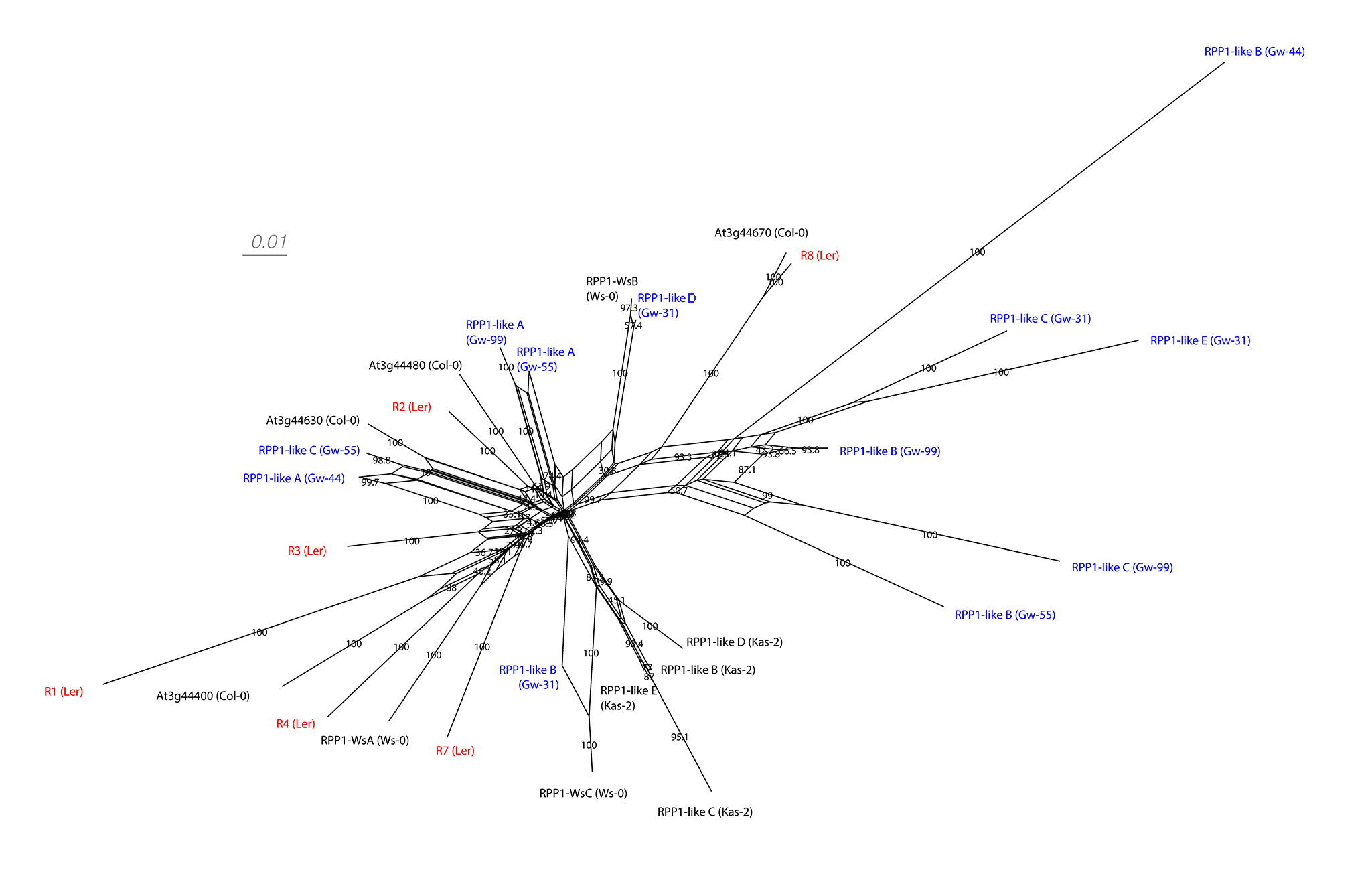

Supplement: S12 Figure — Phylogenetic analysis of RPP1-like genes. Neighbor-net representation of RPP1-like genes in Gw− (blue), Gw+/Ler (red), Col-0 (At3g44400, At3g44480, At3g44630 and At3g44670) and Ws-0 (RPP1-WsA, RPP1-WsB and RPP1-WsC) (black). Parallelograms in the network may indicate recombination events. (TIF) [file pgen.1004848.s012.tif]

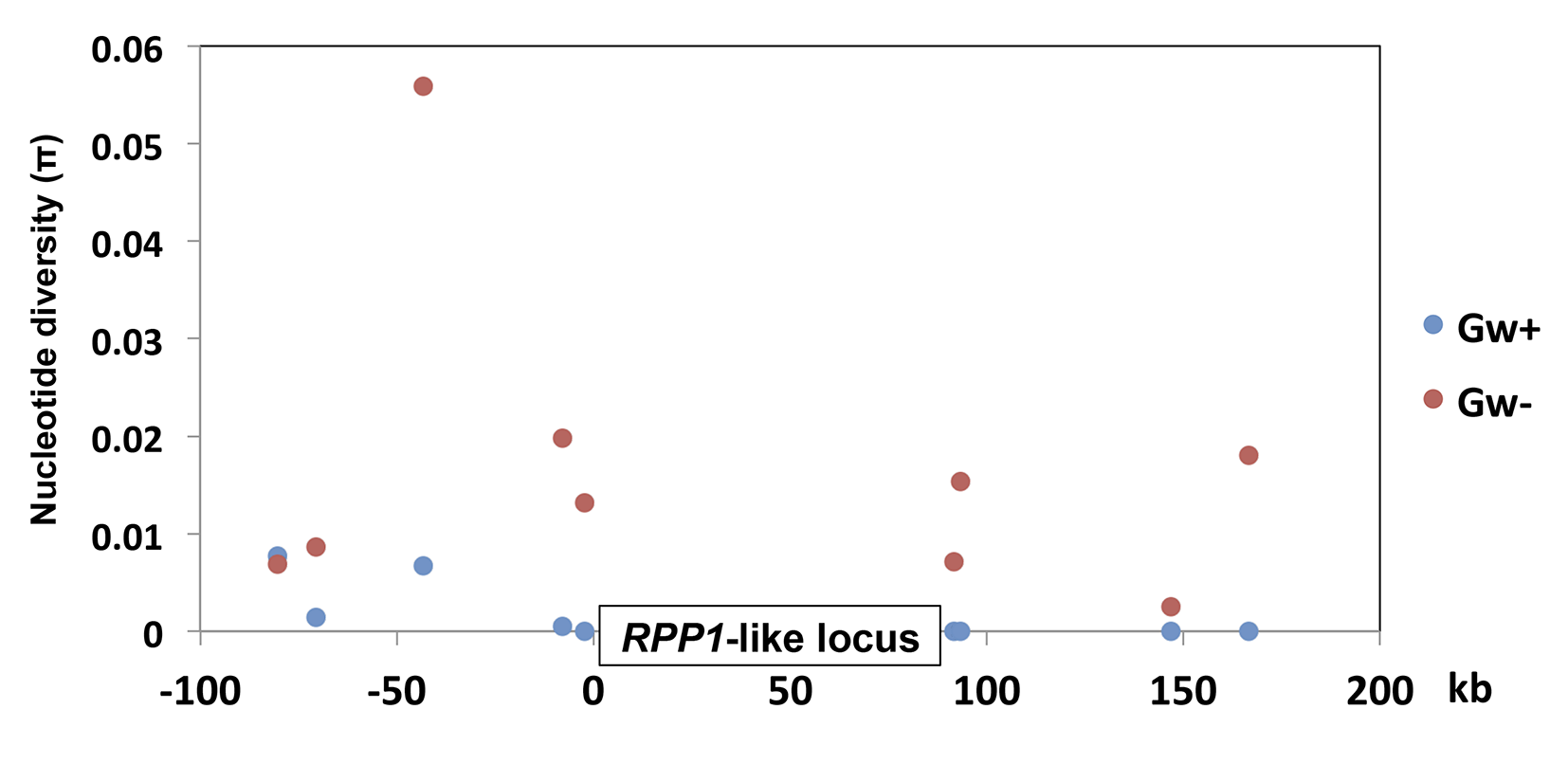

Supplement: S13 Figure — Nucleotide diversity across the RPP1-like locus in Gw+ and Gw−. The data were obtained from sequencing flanking genes in Gw+ (Gw-7, Gw-59, Gw-81, Gw-87, Gw-89, Gw-91, Gw-95, Gw-98, Gw-108, Gw-117, Gw-152 and Gw-159) and Gw− (Gw-2, Gw-19, Gw-23, Gw-31, Gw-44, Gw-55, Gw-69, Gw-99, Gw-119, Gw-140, Gw-144 and Gw-160) accessions at indicated intervals. Lower nucleotide diversity in Gw+ compared to Gw− accessions suggests that recombination is suppressed in accessions carrying the RPP1-like Ler cluster (Gw+). (TIF) [file pgen.1004848.s013.tif]
