# Supplementary material for: Analysis of a Plant Complex Resistance Gene Locus Underlying Immune-Related Hybrid Incompatibility and Its Occurrence in Nature
Source: PLoS Genet. 2014 Dec 11;10(12):e1004848. doi: 10.1371/journal.pgen.1004848 (PMC4263378; doi:10.1371/journal.pgen.1004848)
Supplement: S1 Table — Sequences of amiRNAs and predicted complementarity. (DOCX) [file pgen.1004848.s014.docx]

**Table S1**. Sequences of amiRNAs and predicted complementarity.

| **amiRNA** | **Sequence** | ***RPP1*-like L*er* predicted targets** | **suppression of L*er*/Kas-2 HI** |
| --- | --- | --- | --- |
|  |  |  |  |
| KB209 | UGACACAUAAACUCCAUCGGU | *R2, R7, R8* | yes |
|  |  |  |  |
| KB212 | UACAUUUCAACUGCGAGCGUC | *R2, R4, R5, R7, R8* | yes |
|  |  |  |  |
| KB228 | UAUAUCCGUAAUGAUUGCGGC | none | no |
|  |  |  |  |
