# Supplementary material for: Analysis of a Plant Complex Resistance Gene Locus Underlying Immune-Related Hybrid Incompatibility and Its Occurrence in Nature
Source: PLoS Genet. 2014 Dec 11;10(12):e1004848. doi: 10.1371/journal.pgen.1004848 (PMC4263378; doi:10.1371/journal.pgen.1004848)
Supplement: S2 Table — List and sequences of oligonucleotides. (DOCX) [file pgen.1004848.s015.docx]

**Table S2**. List and sequences of oligonucleotides.

| **Primers for qRT-PCR** | |
| --- | --- |
| R1_Fwd | AAGAGCATGTTGAGAAT |
| R1_Rev | CAATTGCTAGAATGTTCC |
| R2_Fwd | CTTTGGAGGATGTGGCA |
| R2_Rev | CAGCTGTGAGAATGATA |
| R3_Fwd | TGGATTTACGTGAGGAAGAATTG |
| R3_Rev | GAAGTGAAGCAGTTTCTGTCT |
| R4_Fwd | TGCATGTATGTTTGATCGGT |
| R4_Rev | GTCATGATTGCAGCAAATGGA |
| R5_Fwd | TTGCTCAAGTCTGGTGAGAAT |
| R5_Rev | AGCTCAACAAGATTTGAGCAATCA |
| R7_Fwd | GTGGGATATCCATCAAATT |
| R7_Rev | CAATCTCATCGAAACCTTCG |
| R8_Fwd | GCAACTAATCTCGAAGAATTG |
| R8_Rev | AGACTTGAGCACCTTTGGAGATA |
|  |  |
| **Primers for cloning *RPP1*-like L*er* genes** | |
| R1F | TGCAAATGGGATTCAGCATA |
| R1R | ACTGAATTTCTCGGAAGCCA |
| R2F | TGGCTTCCGAGAAATTCAGT |
| R2R | ACAGACCTCAAGGCCAAAGA |
| R3F | GAACAATTGGTCAATCATGGTTAT |
| R3R | TGTCCGAACGAAACAGATCA |
| R4F | GGTGATTGATGCTTGATTGG |
| R4R | GGTGATTGATGCTTGATTGG |
| R5F | GGAGAAGCTGAATGCGGATA |
| R5R | TGCTCAAGGAAGTCAAGCCT |
| R6F | TGTGGACGTGAGGTGTTGTT |
| R6R | TTGGGGGCATGCTTCCTACT |
| R7F | TGCATATGATTTATTCTCGTAGCC |
| R7R | TTTGCAAACGGTTTCGAGTA |
| R8F | AATGTGTGGAAAGCCAGAGG |
| R8R | GCAATCAAGCGCATTACAGA |
| RA | ATGGGTTCTGCAATGAGCTTG |
| RB | ATGGATTCTTCTTTTTTCC |
| RC | ATTGAAGCAGGCAGGCACTT |
|  |  |
| **Primers for sequencing *RPP1*-like L*er* genes** | |
| R1seq_1 | CCTTGGTTCAGTGGCAGCCG |
| R1seq_10 | CAGGCAGGCACTTGTGTCCC |
| R1seq_11 | GACACAAGTGCCTGCCTGCT |
| R1seq_12 | ATCATGCCCCGGTTTACCGC |
| R1seq_2 | TTACCGATCCCAGGCGGTCC |
| R1seq_3 | GGACCGCCTGGGATCGGTAA |
| R1seq_4 | TCTGGCTTGGACTTCCCCCG |
| R1seq_5 | CGGGGGAAGTCCAAGCCAGA |
| R1seq_6 | ACAGGGGGAGATGCAATGGCA |
| R1seq_7 | TGCCATTGCATCTCCCCCTGT |
| R1seq_8 | GCAGGGAGCTCCACAACACG |
| R1seq_9 | CGTGTTGTGGAGCTCCCTGC |
| R2seq_1 | TTGGCTCAGTGGCAGCCGTA |
| R2seq_10 | GGCAGGCACTTGTGTACCGG |
| R2seq_11 | CCGGTACACAAGTGCCTGCC |
| R2seq_12 | GCTCCGTGGAAGTCACCTCCT |
| R2seq_2 | AATCCCAGGCGGTCCCCAAA |
| R2seq_3 | TTTGGGGACCGCCTGGGATT |
| R2seq_6 | TAGGGGGCGATGCAATGGCA |
| R2seq_7 | TGCCATTGCATCGCCCCCTA |
| R2seq_8 | GCGGGGAGCTTCACAACACG |
| R2seq_9 | ACGTGTTGTGAAGCTCCCCG |
| R3seq_1 | CCGAGCTTCCACGGAGCAGA |
| R3seq_10 | GCTCAACTGCAGCTCCGTGA |
| R3seq_11 | CACGGAGCTGCAGTTGAGCAA |
| R3seq_12 | GGCGAGAGATCCGGCGAAGA |
| R3seq_2 | ATCCCAGGCGGTCCCCAAAT |
| R3seq_3 | ATTTGGGGACCGCCTGGGAT |
| R3seq_4 | GCTTGGACATCCCCCGCAAA |
| R3seq_5 | CGGGGGATGTCCAAGCCAGA |
| R4seq_1 | TCCCGAGCTTCCACGGAGCA |
| R4seq_10 | CAGGCACTTGTGTCCCGGGT |
| R4seq_11 | ACCCGGGACACAAGTGCCTG |
| R4seq_12 | GGCGGAAGATCCGGCGAAGA |
| R4seq_2 | TGTTCCCCGGCGATTGTGGC |
| R4seq_3 | GCCACAATCGCCGGGGAACA |
| R4seq_4 | CCCCGCAAAGCGGAGCCTAG |
| R4seq_5 | CTAGGCTCCGCTTTGCGGGG |
| R4seq_8 | GCGGGGAGCTCCACAACACG |
| R4seq_9 | CACGTGTTGTGGAGCTCCCCG |
| R5seq_1 | CTCAGTGGCAGCCGCAGCTT |
| R5seq_2 | AGGCGGTCCCCAAATCCCGA |
| R5seq_3 | GGGACCGCCTGGGATCGGTA |
| R5seq_4 | TCCACGCAGAGCGGAGCCTA |
| R6seq_1 | CCTTGTCGTAGTCGCAGCTGCT |
| R6seq_2 | TGCCCCGTGGAAGCTAGGGA |
| R6seq_3 | TCCCTAGCTTCCACGGGGCA |
| R6seq_4 | TCTCCGGCGATTGTGGCCAC |
| R6seq_5 | TGGCCACAATCGCCGGAGAAC |
| R6seq_6 | GCAGGCAGGCACTTGTGTACCT |
| R6seq_7 | CACGGAGCTGCAGTTGAGCA |
| R7seq_1 | GCTCAGTGGCAGCCGTAGCT |
| R7seq_10 | GGAGGCACTTGTGTCCCGGGT |
| R7seq_11 | GGGGACCGCCTGGGATTGG |
| R7seq_12 | CCCCCGCAAAGCAGAGCCT |
| R7seq_2 | CCCAGGCGGTCCCCAAATCC |
| R7seq_3 | GGATTTGGGGACCGCCTGGG |
| R7seq_4 | TCCCCCGCAAAGCAGAGCCT |
| R7seq_5 | GCGGGGGAAGTCCAAGCCAG |
| R8seq_1 | TGGTTCAGTGGCAGCCGCAG |
| R8seq_10 | CAGGCGGTCCCCAAATCCCG |
| R8seq_2 | AGGCGGTCCCCAAATCCCGA |
| R8seq_3 | CGGGATTTGGGGACCGCCTG |
| R8seq_4 | CAGGGGGCGATGCAATGGCA |
| R8seq_5 | GCCATTGCATCGCCCCCTGT |
| R8seq_7 | CCGGGACACAAGTGCCTGCC |
| R8seq_8 | GGCGAAGAGCTCCGGCGAAG |
| R8seq_9 | GTTCAGTGGCAGCCGCAGCT |
