# Supplementary material for: Analysis of a Plant Complex Resistance Gene Locus Underlying Immune-Related Hybrid Incompatibility and Its Occurrence in Nature
Source: PLoS Genet. 2014 Dec 11;10(12):e1004848. doi: 10.1371/journal.pgen.1004848 (PMC4263378; doi:10.1371/journal.pgen.1004848)
Supplement: S3 Table — Segregation analyses for the occurrence of incompatible phenotypes at 14–16°C in F2 populations derived from the cross of ColRPP1 lines to Kas-2 and Kond. Color scale represents the variation in transgene expression observed between lines. (DOCX) [file pgen.1004848.s016.docx]

**Table S3**. Segregation analyses for the occurrence of incompatible phenotypes at 14- 16 °C in F_2_ populations derived from the cross of Col*^RPP1^* lines to Kas-2 and Kond. Color scale represents the variation in transgene expression observed between lines.

|  |  |  |  |  |
| --- | --- | --- | --- | --- |
|  |  |  |  |  |
| **F_2_ population** | **incompatible plants** | **total nr. plants** | **% incompatible** | **Transgene expression** |
| R1 21.1 x Kas-2 | 0 | 363 | 0.0 |  |
| R1 11.2 x Kas-2 | 0 | 101 | 0.0 |  |
| R1 12.2 x Kas-2 | 0 | 96 | 0.0 |  |
| R1 1.6 x Kas-2 | 0 | 94 | 0.0 |  |
| R1 2.4 x Kas-2 | 0 | 98 | 0.0 |  |
| R1 20.4 x Kas-2 | 0 | 96 | 0.0 |  |
| R1 18.4 x Kas-2 | 0 | 96 | 0.0 |  |
| R1 17.4 x Kas-2 | 0 | 96 | 0.0 |  |
| R1 17.4 x Kond | 0 | 245 | 0.0 |  |
| R1 26.2 x Kas-2 | 0 | 96 | 0.0 |  |
| R1 16.3 x Kas-2 | 0 | 256 | 0.0 |  |
| R1 19.2 x Kas-2 | 0 | 280 | 0.0 |  |
| R1 19.2 x Kond | 0 | 280 | 0.0 |  |
| R2 4.1 x Kas-2 | 0 | 157 | 0.0 |  |
| R2 2.4 x Kas-2 | 0 | 248 | 0.0 |  |
| R2 4b.1 x Kas-2 | 0 | 280 | 0.0 |  |
| R2 12.1 x Kas-2 | 0 | 280 | 0.0 |  |
| R2 1.1 x Kas-2 | 0 | 229 | 0.0 |  |
| R2 1.1 x Kond | 0 | 267 | 0.0 |  |
| R2 14.1 x Kas-2 | 0 | 226 | 0.0 |  |
| R2 14.1 x Kond | 0 | 245 | 0.0 |  |
| R3 24.6 x Kas-2 | 0 | 280 | 0.0 |  |
| R3 24.6 x Kond | 0 | 204 | 0.0 |  |
| R3 25.3 x Kas-2 | 0 | 280 | 0.0 |  |
| R3 14.1 x Kas-2 | 0 | 280 | 0.0 |  |
| R3 21.4 x Kas-2 | 0 | 192 | 0.0 |  |
| R3 27.5 x Kas-2 | 30 | 231 | 13.0 |  |
| R3 12.1 x Kas-2 | 62 | 251 | 24.7 |  |
| R3 12.1 x Kond | 54 | 264 | 20.5 |  |
| R4 22.3 x Kas-2 | 0 | 212 | 0.0 |  |
| R4 15.5 x Kas-2 | 0 | 211 | 0.0 |  |
| R4 33.5 x Kas-2 | 0 | 245 | 0.0 |  |
| R4 23.1 x Kas-2 | 0 | 220 | 0.0 |  |
| R4 10.4 x Kond | 0 | 259 | 0.0 |  |
| R4 25.3 x Kas-2 | 0 | 245 | 0.0 |  |
| R4 25.3 x Kond | 0 | 245 | 0.0 |  |
| R5 5.1 x Kas-2 | 0 | 195 | 0.0 |  |
| R5 4.6 x Kas-2 | 0 | 201 | 0.0 |  |
| R5 1.3 x Kas-2 | 0 | 205 | 0.0 |  |
| R5 6.4 x Kas-2 | 0 | 211 | 0.0 |  |
| R7 37.1 x Kas-2 | 0 | 220 | 0.0 |  |
| R7.1.1 x Kas-2 | 0 | 210 | 0.0 |  |
| R7 1.1 x Kond | 0 | 280 | 0.0 |  |
| R7 2.1 x Kas-2 | 0 | 225 | 0.0 |  |
| R7 35.1 x Kas-2 | 0 | 215 | 0.0 |  |
| R7 12.2 x Kas-2 | 0 | 280 | 0.0 |  |
| R7 3.4 x Kond | 0 | 245 | 0.0 |  |
| R8 10.3 x Kas-2 | 0 | 275 | 0.0 |  |
| R8 10.3 x Kond | 0 | 245 | 0.0 |  |
| R8 17.2 x Kas-2 | 0 | 245 | 0.0 |  |
| R8 17.2 x Kond | 0 | 212 | 0.0 |  |

|  |  | L*er* |  |  |
| --- | --- | --- | --- | --- |
|  |  |  |  |  |
| 0 | | 1 |  | max |
