# Supplementary material for: Analysis of a Plant Complex Resistance Gene Locus Underlying Immune-Related Hybrid Incompatibility and Its Occurrence in Nature
Source: PLoS Genet. 2014 Dec 11;10(12):e1004848. doi: 10.1371/journal.pgen.1004848 (PMC4263378; doi:10.1371/journal.pgen.1004848)
Supplement: S4 Table — List of Arabidospsis thaliana accessions used in this study and presence (+) or absence (−) of RPP1-like Ler haplotype. (DOCX) [file pgen.1004848.s017.docx]

**Table S4**. List of *Arabidospsis thaliana* accessions used in this study and presence (+) or absence (-) of *RPP1*-like L*er* haplotype.

|  |  |  |  |  |  |
| --- | --- | --- | --- | --- | --- |
| **ABRC  Stock number** | **Accession name** | **Latitude** | **Longitude** | **Country** | ***Presence of RPP1-like  Ler haplotype*** |
| CS20 | **L*er*** | 52.733333 | 15.233 | POL | **+** |
| CS28007 | **Aa-0** | 50.9167 | 9.57073 | GER | - |
| CS28013 | **Alst-1** | 54.8 | -2.4333 | UK | - |
| CS28014 | **Amel-1** | 53.448 | 5.73 | NED | - |
| CS28017 | **An-2** | 51.2167 | 4.4 | BEL | - |
| CS28018 | **Ang-0** | 50.3 | 5.3 | BEL | - |
| CS28049 | **Ann-1** | 45.9 | 6.13028 | FRA | - |
| CS28051 | **Arby-1** | 59.4308 | 16.7999 | SWE | - |
| CS28053 | **Ba-1** | 56.5459 | -4.79821 | UK | - |
| CS28054 | **Baa-1** | 51.3333 | 6.1 | NED | - |
| CS28063 | **Be-1** | 49.6803 | 8.6161 | GER | - |
| CS28064 | **Benk-1** | 52 | 5.675 | NED | - |
| CS28090 | **Blh-2** | 48 | 19 | CZE | - |
| CS28091 | **Boot-1** | 54.4 | -3.2667 | UK | - |
| CS28097 | **Bs-2** | 47.5 | 7.5 | SUI | - |
| CS28099 | **Bsch-0** | 40.0167 | 8.6667 | GER | - |
| CS28128 | **Ca-0** | 50.2981 | 8.26607 | GER | - |
| CS28133 | **Cha-0** | 46.0333 | 7.1167 | SUI | - |
| CS28135 | **Chat-1** | 48.0717 | 1.33867 | FRA | - |
| CS28140 | **CIBC-2** | 51.4083 | -0.6383 | UK | - |
| CS28141 | **CIBC-4** | 51.4083 | -0.6383 | UK | - |
| CS28142 | **CIBC-5** | 51.4083 | -0.6383 | UK | - |
| CS28158 | **Cit-0** | 43.3779 | 2.54038 | FRA | - |
| CS28160 | **Cnt-1** | 51.3 | 1.1 | UK | - |
| CS28163 | **Co-2** | 40.12 | -8.25 | POR | - |
| CS28165 | **Co-4** | 40.12 | -8.25 | POR | - |
| CS28181 | **CSHL-5** | 40.8585 | -73.4675 | USA | - |
| CS28193 | **Com-1** | 49.416 | 2.823 | FRA | - |
| CS28200 | **Da-0** | 49.8724 | 8.65081 | GER | - |
| CS28201 | **Da(1)-12** | NA | NA | CZE | - |
| CS28202 | **Db-0** | 50.3055 | 8.324 | GER | - |
| CS28208 | **Di-1** | 47 | 5 | FRA | - |
| CS28210 | **Do-0** | 50.7224 | 8.2372 | GER | - |
| CS28214 | **Dra-2** | 49.4167 | 16.2667 | CZE | - |
| CS28217 | **Ede-1** | 52.0333 | 5.66667 | NED | - |
| CS28236 | **Ep-0** | 50.1721 | 8.38912 | GER | - |
| CS28241 | **Es-0** | 60.1997 | 24.5682 | FIN | - |
| CS28243 | **Est-0** | 58.3 | 25.3 | RUS | - |
| CS28252 | **Fi-1** | 50.5 | 8.0167 | GER | - |
| CS28268 | **Fr-4** | 50.1102 | 8.6822 | GER | - |
| CS28274 | **Ga-2** | 50.3 | 8 | GER | - |
| CS28277 | **Ge-1** | 46.5 | 6.08 | SUI | - |
| CS28279 | **Gel-1** | 51.0167 | 5.86667 | NED | - |
| CS28280 | **Gie-0** | 50.584 | 8.67825 | GER | - |
| CS28282 | **Go-0** | 51.5338 | 9.9355 | GER | - |
| CS28326 | **Gr-5** | 47 | 15.5 | AUT | - |
| CS28332 | **Gu-1** | 50.3 | 8 | GER | - |
| CS28336 | **Ha-0** | 52.3721 | 9.73569 | GER | - |
| CS28343 | **Hau-0** | 55.675 | 12.5686 | DEN | - |
| CS28344 | **Hey-1** | 51.25 | 5.9 | NED | - |
| CS28345 | **Hh-0** | 54.4175 | 9.88682 | GER | - |
| CS28350 | **Hn-0** | 51.3472 | 8.28844 | GER | - |
| CS28364 | **Je-0** | 50.927 | 11.587 | GER | - |
| CS28369 | **Jl-3** | 49.2 | 16.6166 | CZE | - |
| CS28373 | **Jm-1** | 49 | 15 | CZE | - |
| CS28382 | **Kelsterbach-2** | 50.0667 | 8.5333 | GER | - |
| CS28394 | **Kl-5** | 50.95 | 6.9666 | GER | - |
| CS28395 | **Kn-0** | 54.8969 | 23.8924 | LTU | - |
| CS28407 | **KNO-11** | 41.2816 | -86.621 | USA | - |
| CS28419 | **Kr-0** | 51.3317 | 6.55934 | GER | - |
| CS28420 | **Kro-0** | 50.0742 | 8.96617 | GER | - |
| CS28423 | **Krot-2** | 49.631 | 11.5722 | GER | - |
| CS28454 | **Li-3** | 50.3833 | 8.0666 | GER | - |
| CS28457 | **Li-5:2** | 50.3833 | 8.0666 | GER | - |
| CS28459 | **Li-6** | 50.3833 | 8.0666 | GER | - |
| CS28461 | **Li-7** | 50.3833 | 8.0666 | GER | - |
| CS28490 | **Mc-0** | 54.6167 | -2.3 | UK | - |
| CS28492 | **Mh-0** | 50.95 | 7.5 | POL | - |
| CS28495 | **Mnz-0** | 50.001 | 8.26664 | GER | - |
| CS28510 | **N4** | 61.36 | 34.15 | RUS | - |
| CS28513 | **N7** | 61.36 | 34.15 | RUS | - |
| CS28527 | **Nc-1** | 48.6167 | 6.25 | FRA | - |
| CS28550 | **NFC-20** | 51.4083 | -0.6383 | UK | - |
| CS28564 | **No-0** | 51.0581 | 13.2995 | GER | - |
| CS28568 | **Nok-1** | 52.24 | 4.45 | NED | - |
| CS28573 | **Nw-0** | 50.5 | 8.5 | GER | - |
| CS28575 | **Nw-2** | 50.5 | 8.5 | GER | - |
| CS28578 | **Nz1** | -37.7871 | 175.283 | NZL | - |
| CS28580 | **Ob-1** | 50.2 | 8.5833 | GER | - |
| CS28583 | **Old-1** | 53.1667 | 8.2 | GER | - |
| CS28587 | **Or-0** | 50.3827 | 8.01161 | GER | - |
| CS28595 | **Pa-2** | 38.07 | 13.22 | ITA | - |
| CS28108 | **Bu-8** | 50.5 | 9.5 | GER | - |
| CS28613 | **PHW-13** | 51.2878 | 0.0565 | UK | - |
| CS28614 | **PHW-14** | 51.2878 | 0.0565 | UK | - |
| CS28620 | **PHW-20** | 51.2878 | 0.0565 | UK | - |
| CS28622 | **PHW-22** | 51.4167 | -1.7167 | UK | - |
| CS28626 | **PHW-26** | 50.6728 | -3.8404 | UK | - |
| CS28628 | **PHW-28** | 50.35 | -3.5833 | UK | - |
| CS28631 | **PHW-31** | 51.4666 | -3.2 | UK | - |
| CS28633 | **PHW-33** | 52.25 | 4.5667 | NED | - |
| CS28635 | **PHW-35** | 48.6103 | 2.3086 | FRA | - |
| CS22689 | **RRS-10** | 41.5609 | -86.4251 | USA | - |
| CS28636 | **PHW-36** | 48.6103 | 2.3086 | FRA | - |
| CS28637 | **PHW-37** | 48.6103 | 2.3086 | FRA | - |
| CS28640 | **Pla-0** | 41.5 | 2.25 | ESP | - |
| CS28645 | **Pn-0** | 48.0653 | -2.96591 | FRA | - |
| CS28650 | **Pog-0** | 49.2655 | -123.206 | CAN | - |
| CS28651 | **Pr-0** | 50.1448 | 8.60706 | GER | - |
| CS28663 | **Pu2-24** | 49.42 | 16.36 | CZE | - |
| CS28685 | **Rhen-1** | 51.9667 | 5.56667 | NED | - |
| CS28692 | **Rou-0** | 49.4424 | 1.09849 | FRA | - |
| CS28713 | **RRS-7** | 41.5609 | -86.4251 | USA | - |
| CS28720 | **S96** | NA | NA | UNK | - |
| CS28724 | **Sapporo-0** | 43.0553 | 141.346 | JPN | - |
| CS28725 | **Sav-0** | 49.1833 | 15.8833 | CZE | - |
| CS28729 | **Sei-0** | 46.5438 | 11.5614 | ITA | - |
| CS28732 | **Sg-1** | 47.6667 | 9.5 | GER | - |
| CS28734 | **Sh-0** | 51.6832 | 10.2144 | GER | - |
| CS28739 | **Si-0** | 50.8738 | 8.02341 | GER | - |
| CS28743 | **Sp-0** | 52.5339 | 13.181 | GER | - |
| CS28750 | **Ste-0** | 52.6058 | 11.8558 | GER | - |
| CS28758 | **Tha-1** | 52.08 | 4.3 | NED | - |
| CS28759 | **Ting-1** | 56.5 | 14.9 | SWE | - |
| CS28760 | **Tiv-1** | 41.96 | 12.8 | ITA | - |
| CS28779 | **Tscha-1** | 47.0748 | 9.9042 | AUT | - |
| CS28780 | **Tsu-0** | 34.43 | 136.31 | JPN | - |
| CS28786 | **Ty-0** | 56.4278 | -5.23439 | UK | - |
| CS28787 | **Uk-1** | 48.0333 | 7.7667 | GER | - |
| CS28788 | **Uk-2** | 48.0333 | 7.7667 | GER | - |
| CS28795 | **Utrecht** | 52.0918 | 5.1145 | NED | - |
| CS28800 | **Ven-1** | 52.0333 | 5.55 | NED | - |
| CS28804 | **Wa-1** | 52.3 | 21 | POL | - |
| CS28808 | **Wag-3** | 51.9666 | 5.6666 | NED | - |
| CS28809 | **Wag-4** | 51.9666 | 5.6666 | NED | - |
| CS28810 | **Wag-5** | 51.9666 | 5.6666 | NED | - |
| CS28812 | **WAR** | 41.7302 | -71.2825 | USA | - |
| CS28814 | **Wc-2** | 52.6 | 10.0667 | GER | - |
| CS28822 | **Wl-0** | 47.9299 | 10.8134 | GER | - |
| CS28823 | **Ws** | 52.3 | 30 | RUS | - |
| CS28833 | **Wt-3** | 52.3 | 9.3 | GER | - |
| CS28847 | **Zu-1** | 47.3667 | 8.55 | SUI | - |
| CS28848 | **Ors-1** | 44.7203 | 22.3955 | ROU | - |
| CS76086 | **627ME-4Y1** | 42.093 | -86.359 | USA | - |
| CS76083 | **11ME1.32** | 42.093 | -86.359 | USA | - |
| CS76084 | **11PNA4.101** | 42.0945 | -86.3253 | USA | - |
| CS76085 | **328PNA054** | 42.0945 | -86.3253 | USA | - |
| CS76087 | **Ag-0** | 45 | 1.3 | FRA | - |
| CS76088 | **Alc-0** | 40.31 | -3.22 | ESP | - |
| CS76089 | **ALL1-2** | 45.2667 | 1.48333 | FRA | - |
| CS76090 | **ALL1-3** | 45.2667 | 1.48333 | FRA | - |
| CS76091 | **An-1** | 51.2167 | 4.4 | BEL | - |
| CS76092 | **App1-16** | 56.3333 | 15.9667 | SWE | - |
| CS76093 | **Bay1-2** | 56.4 | 12.9 | SWE | - |
| CS76094 | **Bay-0** | 49 | 11 | GER | - |
| CS76095 | **Belmonte-4-94** | 42.1167 | 12.4833 | ITA | - |
| CS76096 | **Bg-2** | 47.6479 | -122.305 | USA | - |
| CS76097 | **Bla-1** | 41.6833 | 2.8 | ESP | - |
| CS76098 | **Blh-1** | 48 | 19 | CZE | - |
| CS76099 | **Bor-1** | 49.4013 | 16.2326 | CZE | - |
| CS76100 | **Bor-4** | 49.4013 | 16.2326 | CZE | - |
| CS76101 | **Br-0** | 49.2 | 16.6166 | CZE | - |
| CS76102 | **Bro1-6** | 56.3 | 16 | SWE | - |
| CS76103 | **Bu-0** | 50.5 | 9.5 | GER | - |
| CS76105 | **Bur-0** | 54.1 | -6.2 | IRL | - |
| CS76106 | **C24** | 41.25 | -8.45 | POR | - |
| CS76107 | **CAM-16** | 48.2667 | -4.58333 | FRA | - |
| CS76108 | **CAM-61** | 48.2667 | -4.58333 | FRA | - |
| CS76109 | **Can-0** | 29.2144 | -13.4811 | ESP | - |
| CS76110 | **Cen-0** | 49 | 0.5 | FRA | - |
| CS76116 | **Cvi-0** | 15.1111 | -23.6167 | CPV | - |
| CS76113 | **Col-0** | 38.3 | -92.3 | USA | - |
| CS76114 | **Ct-1** | 37.3 | 15 | ITA | - |
| CS76115 | **CUR-3** | 45 | 1.75 | FRA | - |
| CS76111 | **CIBC-17** | 51.4083 | -0.6383 | UK | - |
| CS76117 | **Dra3-1** | 55.76 | 14.12 | SWE | - |
| CS76118 | **DraII-1** | 49.4112 | 16.2815 | CZE | - |
| CS76119 | **DraIV 1-14** | 49.4112 | 16.2815 | CZE | - |
| CS76120 | **DraIV 1-5** | 49.4112 | 16.2815 | CZE | - |
| CS76122 | **DraIV 6-16** | 49.4112 | 16.2815 | CZE | - |
| CS76123 | **DraIV 6-35** | 49.4112 | 16.2815 | CZE | - |
| CS76124 | **Duk** | 49.1 | 16.2 | CZE | - |
| CS76125 | **Eden-2** | 62.877 | 18.177 | SWE | - |
| CS76126 | **Edi-0** | 56 | -3 | UK | - |
| CS76127 | **Est-1** | 58.3 | 25.3 | RUS | - |
| CS76128 | **Fab-4** | 63.0165 | 18.3174 | SWE | - |
| CS76129 | **Fei-0** | 40.5 | -8.32 | POR | - |
| CS76130 | **Fja 1-1** | 56.06 | 14.29 | SWE | - |
| CS76131 | **Fja 1-2** | 56.06 | 14.29 | SWE | - |
| CS76132 | **Fja 1-5** | 56.06 | 14.29 | SWE | - |
| CS76133 | **Ga-0** | 50.3 | 8 | GER | - |
| CS76134 | **Gd-1** | 53.5 | 10.5 | GER | - |
| CS76135 | **Ge-0** | 46.5 | 6.08 | SUI | - |
| CS76136 | **Got-7** | 51.5338 | 9.9355 | GER | - |
| CS76137 | **Gr-1** | 47 | 15.5 | AUT | - |
| CS76139 | **Gy-0** | 49 | 2 | FRA | - |
| CS76140 | **Hi-0** | 52 | 5 | NED | - |
| CS76141 | **Hod** | 48.8 | 17.1 | CZE | - |
| CS76142 | **Hov4-1** | 56.1 | 13.74 | SWE | - |
| CS76143 | **Hovdala-2** | NA | NA | Central Asia | - |
| CS76144 | **HR-5** | 51.4083 | -0.6383 | UK | - |
| CS76145 | **Hs-0** | 52.24 | 9.44 | GER | - |
| CS76146 | **HSm** | 49.33 | 15.76 | CZE | - |
| CS76147 | **In-0** | 47.5 | 11.5 | AUT | - |
| CS76148 | **JEA** | 43.6833 | 7.33333 | FRA | - |
| CS76149 | **Ka-0** | 47 | 14 | AUT | - |
| CS76150 | **Kas-1** | 35 | 77 | IND | - |
| CS76151 | **KBS-Mac-8** | 42.405 | -85.398 | USA | - |
| CS76152 | **Kelsterbach-4** | 50.0667 | 8.5333 | GER | - |
| CS76153 | **Kin-0** | 44.46 | -85.37 | USA | - |
| CS76154 | **Kno-18** | 41.2816 | -86.621 | USA | - |
| CS76155 | **Köln** | 51 | 7 | GER | - |
| CS76156 | **Kulturen-1** | 55.705 | 13.196 | SWE | - |
| CS76157 | **LAC-3** | 47.7 | 6.81667 | FRA | - |
| CS76158 | **LAC-5** | 47.7 | 6.81667 | FRA | - |
| CS76159 | **Lc-0** | 57 | -4 | UK | - |
| CS76160 | **LDV-14** | 48.5167 | -4.06667 | FRA | - |
| CS76161 | **LDV-25** | 48.5167 | -4.06667 | FRA | - |
| CS76162 | **LDV-34** | 48.5167 | -4.06667 | FRA | - |
| CS76163 | **LDV-58** | 48.5167 | -4.06667 | FRA | - |
| CS76165 | **LI-OF-095** | 40.7777 | -72.9069 | USA | - |
| CS76166 | **Liarum** | 55.95 | 13.85 | SWE | - |
| CS76167 | **Lillo-1** | 56.1512 | 15.7844 | SWE | - |
| CS76168 | **Lip-0** | 50 | 19.3 | POL | - |
| CS76169 | **Lis-1** | 56 | 14.7 | SWE | - |
| CS76170 | **Lis-2** | 56 | 14.7 | SWE | - |
| CS76171 | **Lisse** | 52.25 | 4.5667 | NED | - |
| CS76172 | **LL-0** | 41.59 | 2.49 | ESP | - |
| CS76173 | **Lm-2** | 48 | 0.5 | FRA | - |
| CS76174 | **Lom1-1** | 56.09 | 13.9 | SWE | - |
| CS76175 | **Lov-5** | 62.801 | 18.079 | SWE | - |
| CS76176 | **Lp2-2** | 49.38 | 16.81 | CZE | - |
| CS76177 | **Lp2-6** | 49.38 | 16.81 | CZE | - |
| CS76121 | **DraIV 1-7** | 49.4112 | 16.2815 | CZE | - |
| CS76179 | **Lz-0** | 46 | 3.3 | FRA | - |
| CS76180 | **Map-42** | 42.166 | -86.412 | USA | - |
| CS76181 | **MIB-15** | 47.3833 | 5.31667 | FRA | - |
| CS76182 | **MIB-22** | 47.3833 | 5.31667 | FRA | - |
| CS76183 | **MIB-28** | 47.3833 | 5.31667 | FRA | - |
| CS76184 | **MIB-84** | 47.3833 | 5.31667 | FRA | - |
| CS76185 | **MNF-Che-2** | 43.5251 | -86.1843 | USA | - |
| CS76186 | **MNF-Jac-32** | 43.5187 | -86.1739 | USA | - |
| CS76187 | **MNF-Pot-48** | 43.595 | -86.2657 | USA | - |
| CS76188 | **MNF-Pot-68** | 43.595 | -86.2657 | USA | - |
| CS76189 | **MOG-37** | 48.6667 | -4.06667 | FRA | - |
| CS76190 | **Mr-0** | 44.15 | 9.65 | ITA | - |
| CS76191 | **Mrk-0** | 49 | 9.3 | GER | - |
| CS76192 | **Mt-0** | 32.34 | 22.46 | LIB | - |
| CS76193 | **Mz-0** | 50.3 | 8.3 | GER | - |
| CS76194 | **N13** | 61.36 | 34.15 | RUS | - |
| CS76195 | **Na-1** | 47.5 | 1.5 | FRA | - |
| CS76197 | **Nd-1** | 50 | 10 | SUI | - |
| CS76198 | **NFA-10** | 51.4083 | -0.6383 | UK | - |
| CS76199 | **NFA-8** | 51.4083 | -0.6383 | UK | - |
| CS76200 | **Omo2-1** | 56.14 | 15.78 | SWE | - |
| CS76201 | **Or-1** | 56.45 | 16.11 | SWE | - |
| CS76202 | **Ost-0** | 60.25 | 18.37 | SWE | - |
| CS76203 | **Oy-0** | 60.23 | 6.13 | NOR | - |
| CS76205 | **PAR-3** | 46.65 | -0.25 | FRA | - |
| CS76104 | **BUI** | 48.3667 | 0.933333 | FRA | - |
| CS76206 | **PAR-4** | 46.65 | -0.25 | FRA | - |
| CS76207 | **PAR-5** | 46.65 | -0.25 | FRA | - |
| CS76208 | **Paw-3** | 42.148 | -86.431 | USA | - |
| CS76209 | **Pent-1** | 43.7623 | -86.3929 | USA | - |
| CS76210 | **Per-1** | 58 | 56.3167 | RUS | - |
| CS76211 | **Petergof** | 59 | 29 | RUS | - |
| CS76213 | **Pna-17** | 42.0945 | -86.3253 | USA | - |
| CS76214 | **Pro-0** | 43.25 | -6 | ESP | - |
| CS76215 | **Pu2-23** | 49.42 | 16.36 | CZE | - |
| CS76216 | **Ra-0** | 46 | 3.3 | FRA | - |
| CS76217 | **Rak-2** | 49 | 16 | CZE | - |
| CS76218 | **Ren-1** | 48.5 | -1.41 | FRA | - |
| CS76219 | **Rev-2** | 55.7 | 13.4 | SWE | - |
| CS76220 | **Rmx-A180** | 42.036 | -86.511 | USA | - |
| CS76221 | **ROM-1** | 45.5333 | 4.85 | FRA | - |
| CS76222 | **Rsch-4** | 56.3 | 34 | RUS | - |
| CS76223 | **Sanna-2** | 62.69 | 18 | SWE | - |
| CS76224 | **Sap-0** | 49.49 | 14.24 | CZE | - |
| CS76225 | **Sav-0** | 49.1833 | 15.8833 | CZE | - |
| CS76226 | **Se-0** | 38.3333 | -3.53333 | ESP | - |
| CS76227 | **Shahdara** | 38.35 | 68.48 | TJK | - |
| CS76228 | **SLSP-30** | 43.665 | -86.496 | USA | - |
| CS76229 | **Sparta-1** | 55.7097 | 13.0489 | SWE | - |
| CS76230 | **Sq-8** | 51.4083 | -0.6383 | UK | - |
| CS76231 | **St-0** | 59 | 18 | SWE | - |
| CS76232 | **Ste-3** | 42.03 | -86.514 | USA | - |
| CS76233 | **T1040** | 55.6494 | 13.2147 | SWE | - |
| CS76234 | **T1060** | 55.6472 | 13.2225 | SWE | - |
| CS76235 | **T1080** | 55.6561 | 13.2178 | SWE | - |
| CS76236 | **T1110** | 55.6 | 13.2 | SWE | - |
| CS76238 | **T510** | 55.7936 | 13.1233 | SWE | - |
| CS76239 | **T540** | 55.7967 | 13.1044 | SWE | - |
| CS76240 | **T620** | 55.7 | 13.2 | SWE | - |
| CS76254 | **TOU-A1-12** | 46.6667 | 4.11667 | FRA | - |
| CS76242 | **Ta-0** | 49.5 | 14.5 | CZE | - |
| CS76243 | **Tad01** | 62.8714 | 18.3447 | SWE | - |
| CS76244 | **Tamm-2** | 60 | 23.5 | FIN | - |
| CS76245 | **TDr-1** | 55.7683 | 14.1386 | SWE | - |
| CS76257 | **TOU-A1-67** | 46.6667 | 4.11667 | FRA | - |
| CS76247 | **TDr-18** | 55.7714 | 14.1208 | SWE | - |
| CS76248 | **TDr-3** | 55.7686 | 14.1381 | SWE | - |
| CS76249 | **TDr-8** | 55.7706 | 14.1342 | SWE | - |
| CS76250 | **Tomegap-2** | 55.7 | 13.2 | SWE | - |
| CS76251 | **Tottarp-2** | 55.95 | 13.85 | SWE | - |
| CS76252 | **TOU-A1-115** | 46.6667 | 4.11667 | FRA | - |
| CS76253 | **TOU-A1-116** | 46.6667 | 4.11667 | FRA | - |
| CS76212 | **PHW-34** | 48.6103 | 2.3086 | FRA | - |
| CS76256 | **TOU-A1-62** | 46.6667 | 4.11667 | FRA | - |
| CS76258 | **TOU-A1-96** | 46.6667 | 4.11667 | FRA | - |
| CS76259 | **TOU-C-3** | 46.6667 | 4.11667 | FRA | - |
| CS76260 | **TOU-E-11** | 46.6667 | 4.11667 | FRA | - |
| CS76261 | **TOU-H-12** | 46.6667 | 4.11667 | FRA | - |
| CS76262 | **TOU-H-13** | 46.6667 | 4.11667 | FRA | - |
| CS76263 | **TOU-I-17** | 46.6667 | 4.11667 | FRA | - |
| CS76264 | **TOU-I-2** | 46.6667 | 4.11667 | FRA | - |
| CS76265 | **TOU-I-6** | 46.6667 | 4.11667 | FRA | - |
| CS76266 | **TOU-J-3** | 46.6667 | 4.11667 | FRA | - |
| CS76267 | **TOU-K-3** | 46.6667 | 4.11667 | FRA | - |
| CS76268 | **Ts-1** | 41.7194 | 2.93056 | ESP | - |
| CS76269 | **UduI 1-34** | 49.2771 | 16.6314 | CZE | - |
| CS76270 | **UKID101** | 53.2 | -1.4 | UK | - |
| CS76138 | **Gul1-2** | 56.3 | 16 | SWE | - |
| CS76272 | **UKID37** | 51.3 | 1.1 | UK | - |
| CS76273 | **UKID48** | 54.7 | -2.7 | UK | - |
| CS76274 | **UKID80** | 54.7 | -2.9 | UK | - |
| CS76275 | **UKNW06-059** | 54.4 | -3 | UK | - |
| CS76276 | **UKNW06-060** | 54.4 | -3 | UK | - |
| CS76277 | **UKNW06-386** | 54.6 | -3.1 | UK | - |
| CS76278 | **UKNW06-436** | 54.7 | -3.4 | UK | - |
| CS76279 | **UKNW06-460** | 54.7 | -3.4 | UK | - |
| CS76280 | **UKSE06-062** | 51.3 | 0.5 | UK | - |
| CS76281 | **UKSE06-192** | 51.3 | 0.5 | UK | - |
| CS76282 | **UKSE06-272** | 51.3 | 0.4 | UK | - |
| CS76283 | **UKSE06-278** | 51.3 | 0.4 | UK | - |
| CS76284 | **UKSE06-349** | 51.3 | 0.4 | UK | - |
| CS76285 | **UKSE06-351** | 51.3 | 0.4 | UK | - |
| CS76286 | **UKSE06-414** | 51.3 | 0.4 | UK | - |
| CS76287 | **UKSE06-429** | 51.3 | 0.4 | UK | - |
| CS76288 | **UKSE06-466** | 51.2 | 0.4 | UK | - |
| CS76289 | **UKSE06-482** | 51.2 | 0.6 | UK | - |
| CS76290 | **UKSE06-520** | 51.3 | 1.1 | UK | - |
| CS76291 | **UKSE06-628** | 51.1 | 0.4 | UK | - |
| CS76292 | **UKSW06-202** | 50.4 | -4.9 | UK | - |
| CS76293 | **Ull2-3** | 56.0648 | 13.9707 | SWE | - |
| CS76296 | **Uod-7** | 48.3 | 14.45 | AUT | - |
| CS76297 | **Van-0** | 49.3 | -123 | CAN | - |
| CS76298 | **Var2-1** | 55.58 | 14.334 | SWE | - |
| CS76299 | **VOU-1** | 46.65 | 0.166667 | FRA | - |
| CS76300 | **VOU-2** | 46.65 | 0.166667 | FRA | - |
| CS76301 | **Wei-0** | 47.25 | 8.26 | SUI | - |
| CS76303 | **Ws-0** | 52.3 | 30 | RUS | - |
| CS76304 | **Wt-5** | 52.3 | 9.3 | GER | - |
| CS76305 | **Yo-0** | 37.45 | -119.35 | USA | - |
| CS76306 | **Zdr-6** | 49.3853 | 16.2544 | CZE | - |
| CS76307 | **ZdrI 2-24** | 49.3853 | 16.2544 | CZE | - |
| CS76308 | **ZdrI 2-25** | 49.3853 | 16.2544 | CZE | - |
