# Supplementary material for: Analysis of a Plant Complex Resistance Gene Locus Underlying Immune-Related Hybrid Incompatibility and Its Occurrence in Nature
Source: PLoS Genet. 2014 Dec 11;10(12):e1004848. doi: 10.1371/journal.pgen.1004848 (PMC4263378; doi:10.1371/journal.pgen.1004848)
Supplement: S5 Table — Segregation analyses for the occurrence of incompatible phenotypes at 14–16°C in F2 populations derived from the cross of Gw+ and Gw− accessions to Kas-2 and Kond. (DOCX) [file pgen.1004848.s018.docx]

**Table S5.** Segregation analyses for the occurrence of incompatible phenotypes at 14- 16 °C in F_2_ populations derived from the cross of Gw^+^ and Gw^-^ accessions to Kas-2 and Kond.

| **F_2_ Population** | **Mother accession** | | **Father accession** | **No. incompatible plants in F_2_** | **Total F_2_ plants scored** | **% incompatible plants** | **incompatible F2 plants** | | **Genetic architecture of incompatibility** | **Pvalue of Chi² tested** |
| --- | --- | --- | --- | --- | --- | --- | --- | --- | --- | --- |
|  | **Accession** | **Type** |  |  |  |  | ***RPP1*-like alleles** | ***SRF3***  **alleles** |  |  |
| Gw-7 x Kas-2 | Gw-7 | Gw+ | Kas-2 | 8 | 251 | 3.19 | Ler/Ler | Kas-2/Kas-2 | 2 dominant + 2 recessive | 0.78 |
| Gw-80 x Kas-2 | Gw-80 | Gw+ | Kas-2 | 7 | 232 | 3.02 | Ler/Ler | Kas-2/Kas-2 | 2 dominant + 2 recessive | 0.69 |
| Gw-81 x Kas-2 | Gw-81 | Gw+ | Kas-2 | 7 | 269 | 2.60 | Ler/Ler | Kas-2/Kas-2 | 2 dominant + 2 recessive | 0.42 |
| Gw-89 x Kas-2 | Gw-89 | Gw+ | Kas-2 | 8 | 250 | 3.20 | Ler/Ler | Kas-2/Kas-2 | 2 dominant + 2 recessive | 0.79 |
| Gw-91 x Kas-2 | Gw-91 | Gw+ | Kas-2 | 5 | 253 | 1.98 | Ler/Ler | Kas-2/Kas-2 | 3 recessive | 0.60 |
| Gw-92 x Kas-2 | Gw-92 | Gw+ | Kas-2 | 6 | 259 | 2.32 | Ler/Ler | Kas-2/Kas-2 | 3 recessive | 0.33 |
| Gw-108 x Kas-2 | Gw-108 | Gw+ | Kas-2 | 5 | 268 | 1.87 | Ler/Ler | Kas-2/Kas-2 | 3 recessive | 0.69 |
| Gw-117 x Kas-2 | Gw-117 | Gw+ | Kas-2 | 6 | 184 | 3.26 | Ler/Ler | Kas-2/Kas-2 | 2 dominant + 2 recessive | 0.85 |
| Gw-152 x Kas-2 | Gw-152 | Gw+ | Kas-2 | 9 | 211 | 4.27 | Ler/Ler | Kas-2/Kas-2 | 1 dominant + 2 recessive | 0.78 |
| Gw-59 x Kond | Gw-59 | Gw+ | Kond | 13 | 268 | 4.85 | Ler/Ler | Kond/Kond | 1 dominant + 2 recessive | 0.90 |
| Gw-87 x Kond | Gw-87 | Gw+ | Kond | 6 | 269 | 2.23 | Ler/Ler | Kond/Kond | 3 recessive | 0.38 |
| Gw-89 x Kond | Gw-89 | Gw+ | Kond | 6 | 271 | 2.21 | Ler/Ler | Kond/Kond | 3 recessive | 0.39 |
| Gw-98 x Kond | Gw-98 | Gw+ | Kond | 10 | 273 | 3.66 | Ler/Ler | Kond/Kond | 2 dominant + 2 recessive | 0.90 |
| Gw-31 x Kas-2 | Gw-31 | Gw- | Kas-2 | 0 | 234 | 0.00 | - | - | Compatible | - |
| Gw-99 x Kond | Gw-99 | Gw- | Kond | 0 | 245 | 0.00 | - | - | Compatible | - |
| Gw-160 x Kas-2 | Gw-160 | Gw- | Kas-2 | 0 | 239 | 0.00 | - | - | Compatible | - |
